# Characterization of human stem cell-derived hepatic stellate cells and liver sinusoidal endothelial cells during extended *in vitro* culture

**DOI:** 10.3389/fbioe.2023.1223737

**Published:** 2023-07-25

**Authors:** Ingrid Wilhelmsen, Mikel Amirola Martinez, Justyna Stokowiec, Chencheng Wang, Aleksandra Aizenshtadt, Stefan Krauss

**Affiliations:** ^1^ Department of Immunology and Transfusion Medicine, Oslo University Hospital, Oslo, Norway; ^2^ Hybrid Technology Hub—Centre of Excellence, Institute of Basic Medical Sciences, University of Oslo, Oslo, Norway

**Keywords:** co-culture, hepatic stellate (Ito) cell (HSC), human pluripotent stem cell (hPSC), *in vitro*, liver sinusoidal endothelial cell (LSEC), Notch inhibition, stem cell differentiation

## Abstract

**Background:** There is a significant need for predictive and stable *in vitro* human liver representations for disease modeling and drug testing. Hepatic stellate cells (HSCs) and liver sinusoidal endothelial cells (LSECs) are important non-parenchymal cell components of the liver and are hence of relevance in a variety of disease models, including hepatic fibrosis. Pluripotent stem cell- (PSC-) derived HSCs (scHSCs) and LSECs (scLSECs) offer an attractive alternative to primary human material; yet, the suitability of scHSCs and scLSECs for extended *in vitro* modeling has not been characterized.

**Methods:** In this study, we describe the phenotypic and functional development of scHSCs and scLSECs during 14 days of 2D *in vitro* culture. Cell-specific phenotypes were evaluated by cell morphology, immunofluorescence, and gene- and protein expression. Functionality was assessed in scHSCs by their capacity for intracellular storage of vitamin A and response to pro-fibrotic stimuli induced by TGF-β. scLSECs were evaluated by nitric oxide- and factor VIII secretion as well as endocytic uptake of bioparticles and acetylated low-density lipoprotein. Notch pathway inhibition and co-culturing scHSCs and scLSECs were separately tested as options for enhancing long-term stability and maturation of the cells.

**Results and Conclusion:** Both scHSCs and scLSECs exhibited a post-differentiation cell type-specific phenotype and functionality but deteriorated during extended culture with PSC line-dependent variability. Therefore, the choice of PSC line and experimental timeframe is crucial when designing *in vitro* platforms involving scHSCs and scLSECs. Notch inhibition modestly improved long-term monoculture in a cell line-dependent manner, while co-culturing scHSCs and scLSECs provides a strategy to enhance phenotypic and functional stability.

## Introduction

Hepatic homeostasis is influenced by an interplay between hepatocytes and non-parenchymal cells (NPCs) (Ramachandran et al., 2019; Wallace et al., 2022), including hepatic stellate cells (HSCs) and liver sinusoidal endothelial cells (LSECs). In the homeostatic liver, HSCs exist in a non-proliferative, quiescent state in the subendothelial space of Disse which is located between the LSEC and hepatocytes. Here they exert various functions including storage of vitamin A in cytoplasmic lipid droplets (Tsuchida and Friedman, 2017; Kitto and Henderson, 2021). LSECs are specialized endothelial cells that line the liver sinusoid which enables the selective transport of nutrients and scavenger functions (Bhandari et al., 2021).

Liver diseases are a major health burden and were estimated to account for 2.2% of deaths worldwide in 2016 (Cheemerla and Balakrishnan, 2021). Upon liver injury, HSCs transdifferentiate to an activated extracellular matrix-secreting myofibroblast- (MF-) like phenotype (Tsuchida and Friedman, 2017). During chronic liver inflammation, activated HSCs constitute the main source of the MFs responsible for fibrotic development (Mederacke et al., 2013; Iwaisako et al., 2014; Distler et al., 2019). LSECs also contribute to the fibrotic niche formation by losing their fenestrated phenotype, a phenomenon known as LSEC dedifferentiation (Xie et al., 2012).

These functions make HSCs and LSECs essential parts of predictive *in vitro* liver models for studying a plethora of disease conditions, including fibrosis (Thompson and Takebe, 2021). However, the use of primary HSCs (pHSCs) and LSECs (pLSECs) from human donors is limited by availability, batch variations, and phenotypical and functional changes during isolation and *in vitro* cultivation (Zeilinger et al., 2016). Specifically, pHSCs are easily activated, e.g., by the stiffness of the culture substrate, making it challenging to model progressive conditions like fibrosis where the activation process of HSCs is central to the pathogenesis (Sancho-Bru et al., 2005; Sakai and Yoshimura, 2021). For pLSECs, no *in vitro* model known to the authors allow the preservation of their fenestrated phenotype, hampering their functionality.

Pluripotent stem cells (PSCs), including human induced PSCs (hiPSC) and human embryonic stem cells (hESC), differentiated toward HSCs and LSECs are a promising alternative source to primary NPCs. Recent studies have established differentiation protocols for both stem cell-derived HSCs (scHSCs) (Coll et al., 2018; Koui et al., 2021; Vallverdú et al., 2021) and stem cell-derived LSECs (scLSECs) (Gage et al., 2020). However, the phenotypic and functional stability of the PSC-derived NPCs during long-term *in vitro* culture as well as PSC line-dependent variations have not been evaluated. Hence, a systematic approach to assess PSC-derived NPCs for *in vitro* liver- and disease modeling is needed.

Fibrosis is currently irreversible, calling for the development of predictive human *in vitro* models for anti-fibrotic drug discovery (Marcellin and Kutala, 2018; Asrani et al., 2019). The transforming growth factor-beta (TGF-β) signaling pathway is critical in liver fibrogenesis both *in vivo* (Fabregat et al., 2016; Distler et al., 2019; Kumar et al., 2021) and *in vitro* (Aimaiti et al., 2018; Coll et al., 2018; Aimaiti et al., 2019; Koui et al., 2021; Vallverdú et al., 2021), largely due to its involvement in HSC activation (Fabregat et al., 2016; Dewidar et al, 2019). Several studies have demonstrated reciprocal activation of the TGF-β signaling pathway and the Notch signaling pathway during fibrotic development, (Wang et al., 2017; Aimaiti et al., 2018; Aimaiti et al., 2019). In accordance, the inhibition of the Notch pathway by a γ-secretase inhibitor has been shown to attenuate steatosis and hepatic fibrosis in mouse models (Chen et al., 2012; Wang et al., 2017; Fang et al., 2022). Similarly, Notch activation has been linked to the dedifferentiation of LSECs (Duan et al., 2018). Notch inhibition has not been explored in PSC-derived NPC *in vitro* fibrosis models.

In this study, we established scHSC and scLSEC differentiation from several PSC lines, characterized their phenotype and functionality, evaluated changes during long-term *in vitro* culture as individual cell cultures and in a scHSC and scLSEC co-culture, and assayed their responsiveness to Notch inhibition and TGF-β challenge.

## Methods

### Culturing and maintenance of PSCs

Human pluripotent stem cells (sc_1: H1, WiCell Research Institute; sc_2: WTC-11, Corelli Institute for Medical Research; sc_3: WTSli28-A, Wellcome Trust Sanger Institute and sc_4: WTSli013-A, Wellcome Trust Sanger Institute) were cultured in E8 media (Thermo Fisher Scientific, cell lines sc_2, sc_3 and sc_4) or mTeSR medium (StemCell Technologies, 85,850, cell line sc_1) on plates coated with 1% (v/v) Geltrex (Thermo Fisher Scientific) in a humidified 37°C, 5% CO_2_ incubator. The culture medium of PSCs was changed every 24 h. Cells were passaged using 5 µM EDTA (Thermo Fisher Scientific) in DPBS (Thermo Fisher Scientific) and replated as small clumps at a dilution of 1:3–1:5. Quality control including flow cytometry, RT-qPCR, and immunofluorescence imaging for pluripotency markers was performed.

### scHSC differentiation

scHSCs were generated following a modified version of published approaches to scHSC 2D culture differentiation (Coll et al., 2018; Vallverdú et al., 2021).

**FIGURE 1:**
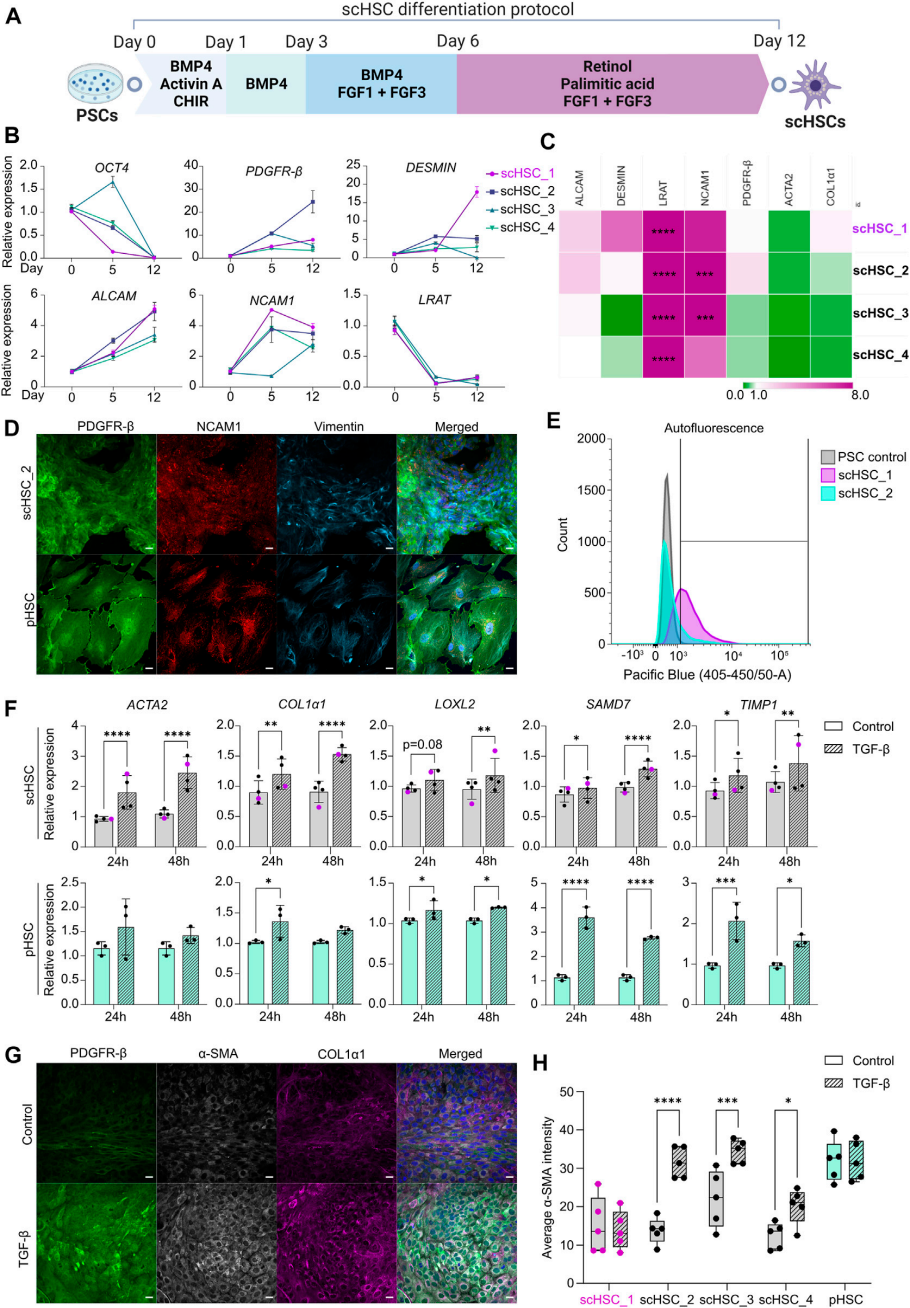
Generation and Characterization of PSC-derived scHSCs. **(A)** Schematic representation of the 12-day scHSC differentiation protocol. **(B)** Gene expression of the pluripotency marker *OCT4* and HSC markers *PDGFR-β, DESMIN, ALCAM, NCAM1* and *LRAT* from n = 4 independent differentiations. Relative expression was normalized to day 0 (PSCs) of the differentiation protocol. N = 3 technical replicates. **(C)** Gene expression of HSC markers from scHSCs at the end of the differentiation protocol normalized to a sample of human pHSCs harvested before culturing. *LRAT* was normalized to pHSCs after 5 days in culture as the *LRAT* level was undetectable in pHSCs before culturing. N = 3 technical replicates. **(D)** Representative immunofluorescence confocal images of PDGFR-β, NCAM1, and Vimentin in scHSC_2 at the end of the differentiation protocol and pHSCs after 5 days in culture. All images were captured using a confocal laser scanning microscope. Scale bars: 40 µm. **(E)** Histograms of flow cytometry analysis of scHSC_1 and scHSC_2. The right-shift indicates autofluorescence at 405 nm, an indirect measure of vitamin A. PSC control: Undifferentiated hESCs. N = 2 technical replicates. **(F)** Gene expression of fibrosis-associated markers in scHSCs and pHSCs after 24 and 48 h of treatment with TGF-β. Expression values were normalized to the control samples. pHSC samples were harvested on day 5. scHSCs: n = 4 independent differentiations, N = 3 technical replicates per differentiation. pHSCs: n = 1 donor, N = 3 technical replicates. **(G)** Representative immunofluorescence confocal images of PDGFR-β, α-SMA, and COL1α1 in scHSC_2. The cells were either treated or not treated with TGF-β before fixation. Scale bars: 40 µm. **(H)** Quantification of the average integral α-SMA intensity within PDGFR-β positive areas of immunofluorescence confocal images, comparing cells either treated or not treated with TGF-β. pHSC samples were harvested on day 5. N = 5 images of independent fields. Data from scHSC_1 (hESC-derived) is colored purple or shown as purple dots throughout the figure. See also Supplementary Figure S1.

Human PSCs were seeded as single cells in a 1:4 ratio on plates coated with 1% (v/v) GelTrex matrix (Thermo Fisher Scientific). hESCs and hiPSCs were cultured in mTeSR medium (STEMCELL Technologies) and E8 medium (Gibco™) respectively supplemented with 10 µM Rock inhibitor (STEMCELL Technologies) the first 24 h after seeding. The scHSC differentiation protocol was initiated at approximately 40% confluency, usually 2 days after seeding. The composition of the scHSC differentiation medium is provided in Supplementary Table S1 and a diagram of the protocol is presented in Figure 1A. The differentiation medium was changed every 48 h. The scHSCs were passaged on day 5 during the differentiation and 10 µM Rock inhibitor was consequently added to the differentiation medium for 24 h. For the passaging, Accutase (Gibco™) was used for detachment and the cells were plated as single cells in a ratio of 1:2–1:3 on plates coated with 1% (v/v) GelTrex matrix.

### scLSEC differentiation

LSEC were generated following a modification of a published protocol (Gage et al., 2020) adapted to the 2D culture.

**FIGURE 2:**
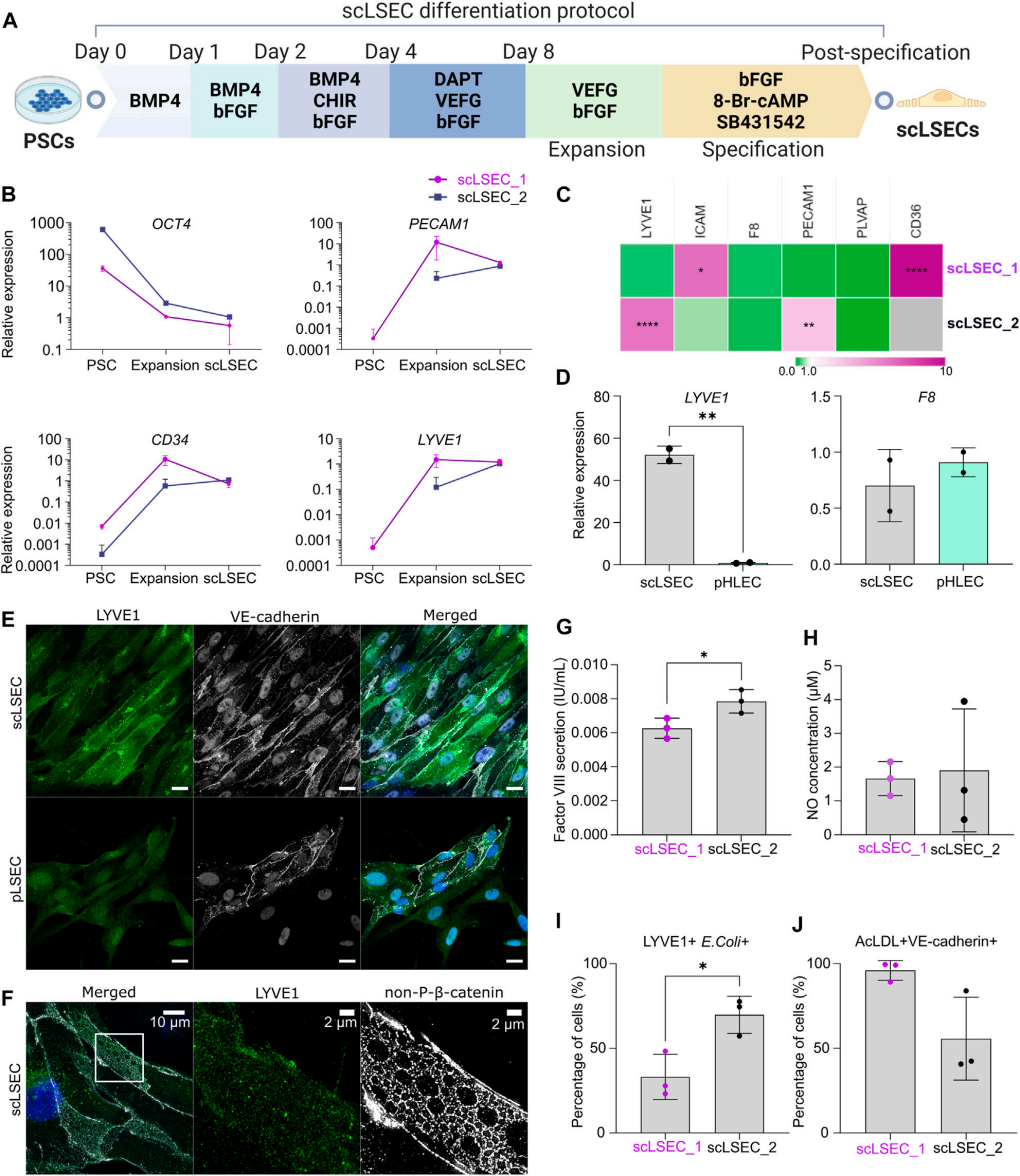
Generation and characterization of PSC-derived scLSECs. **(A)** Schematic depiction of the differentiation protocol from PSC towards scLSEC. **(B)** Gene expression of *OCT4, PECAM1, CD34,* and *LYVE1* during the differentiation protocol (PSC: Pluripotent stem cells, scLSEC: scLSECs at day 0 post-specification). The *y*-axis is logarithmic in scale. Expression values are normalized to scLSECs. N = 3 technical replicates. **(C)** Gene expression of LSEC markers from scLSECs at the end of the differentiation protocol normalized to a sample of human pLSECs harvested before culturing. Gray color indicates no detectable expression. N = 3 technical replicates. **(D)** Gene expression of *LYVE1* and *F8* in scLSEC_2 and benchmark human primary pooled hepatic endothelial cells (pHLEC). Expression values were normalized to the pHLEC samples. N = 2 technical replicates. **(E)** Representative immunofluorescence confocal images of LYVE1 and VE-cadherin in scLSEC_2 and pLSECs after 5 days in culture. All images were captured using a confocal laser scanning microscope. Scale bars: 20 µm. **(F)** Representative immunofluorescence structure illumination microscope (SIM) images of LYVE1 and non-phosphorylated β-catenin (non-P-β-catenin) in a magnified scLSEC_2 cell on day 5 post-differentiation. Scale bars lengths are indicated in the pictures. **(G)** Total coagulation factor VIII concentration in the cell culture medium of scLSECs. N = 3 technical replicates. **(H)** Total nitric oxide (NO) concentration in the cell culture medium of scLSECs at the end of the differentiation protocol. N = 3 technical replicates. **(I)** Percentage of scLSECs positive for LYVE1 and labeled *E. coli* bioparticles as measured by flow cytometry. N = 3 technical replicates. **(J)** Percentage of scLSECs positive for VE-cadherin and labeled acetylated low-density lipoproteins (AcLDL) as measured by flow cytometry. N = 3 technical replicates. Data from scLSEC_1 (hESC-derived) are colored purple or shown as purple dots throughout the. See also Supplementary Figure S2.

PSCs were seeded as small colonies in a 1:10 ratio on plates coated with a 2.5% (v/v) Geltrex matrix (Thermo Fisher Scientific). Cells were incubated for 24 h after seeding in stem cell medium (E8), supplemented with 10 µM Rock inhibitor (STEMCELL Technologies). The composition of the scLSEC differentiation medium is provided in Supplementary Table S1 and a diagram of the protocol is presented in Figure 2A. The differentiation medium was changed every 48 h. At day 8, CD34^+^ cells were selected from the total cell population using anti-CD34 conjugated Dynabeads (Thermo Fisher) and a Dynamag 15 mL tube magnet. Selected cells were then plated on a 12-well plate coated with Geltrex 2.5% (v/v), at a high cell concentration. Cells were cultured and expanded for 3 to 4 passages on 1:3 surface ratios until the necessary number of cells was obtained. Cells were detached for passaging with Trypsin/EDTA solution for approximately 5 min.

scLSEC specification was performed for 5 days in specification medium (see Supplementary Table S1) on fully confluent wells. The differentiation and cultivation of scLSECs was performed under hypoxic conditions (5% O_2_).

### Culture of primary human HSCs, LSECs, and LEC

Commercially available human pHSCs (iXCells Biotechnologies), pLSECs (Axol Bioscience), and primary liver-derived endothelial cells (pHLECs, mixed population) (Lonza) were cultured according to the manufacturer’s instructions. An aliquot of the pHSCs and pLSECs was collected before plating for downstream transcriptomic analysis. Commercial Stellate Cell Growth Medium (iXCells Biotechnologies) and Sinusoidal Endothelial Growth Medium (Axol Bioscience) were used for the initial plating of the pHSCs and pLSECs respectively. For the duration of the long-term culture, pHSCs were grown in HSC expansion medium and pLSECs were grown in LSEC expansion medium (Supplementary Table S1). pHLECs were grown in Endothelial Cell Growth Medium-2 (Lonza).

### Long-term culture of scHSCs and scLSECs

scHSCs were passaged for the long-term culture experiment at the end of the differentiation protocol. The scHSCs were first incubated on ice in Cell Recovery Solution (Corning) for 30 min following detachment by Trypsin-EDTA (Gibco). The cells were then seeded as a single cell suspension at a density of 73,000 cells/cm^2^ and incubated for 1 h at 37°C in DMEM/F12 medium with GelTrex diluted 1:50. The scHSCs were then incubated in HSC expansion medium (see Supplementary Table S1) supplemented with 10 µM Rock inhibitor for 24 h.

scHSCs and scLSECs were cultured in HSC and LSEC expansion medium respectively (see Supplementary Table S1) for the duration of the long-term culture, with or without 5 µM DAPT (Tocris). The medium was changed every 48 h.

### *In vitro* modeling of scHSC- and pHSC activation

Activation modeling of pHSCs and scHSCs was performed by incubation in HSC expansion medium supplemented with 25 ng/mL TGF-β (Abcam) (Coll et al., 2018). The incubation time was 24 h if not otherwise stated. The cells and culture medium were then harvested for downstream analysis. All cells were washed once with room temperature DMEM/F-12 medium before treatment with TGF-β to avoid potential cross-reactions with previous treatments.

### Co-culture of scHSCs and scLSECs

scHSCs were plated in the wells of transwell plates (Corning) while scLSECs were plated in the transwell inserts. The co-culture was initiated by transferring the inserts to the transwells and the cells were incubated under hypoxic conditions (5% O_2_) for the duration of the co-culture. The co-culture medium is specified in Supplementary Table S1 and was changed every 48 h.

### Analysis of gene expression by RNA extraction and real-time quantitative polymerase chain reaction (RT-qPCR)

Cultured cells were snap frozen at −80°C after detachment with Trypsin-EDTA until total RNA isolation was performed by using either TRIzol™ Reagent (Invitrogen, primarily used when the expected RNA yield was high) or RNeasy Mini Kit (Qiagen, primarily used when the expected RNA yield was low). The total RNA isolate was reversely transcribed using a High-Capacity cDNA Reverse Transcription Kit (Applied Biosystems). Gene expression of selected genes was then determined by RT-qPCR analysis on a ViiA 7 (Thermo Fisher Scientific) thermocycler using TaqMan probes (see “Supplementary Table S3”) and TaqMan Gene Expression Master Mix (Thermo Fisher Scientific). Cell pellets, RNA- and cDNA samples were kept on ice during processing and analysis. RNA was stored at −80°C and cDNA was stored at −20°C. For scHSCs and pHSCs, *GAPDH* was used as the housekeeping gene, and for scLSECs, *TBP* was used as the housekeeping gene.

### Measurement of procollagen type I C-peptide secretion

scHSCs and pHSCs were either treated or not treated with 25 ng/mL TGF-β. The conditioned medium was collected after 24 h and stored at −80°C until the assay was performed. The concentration of Procollagen type I in the samples was detected and measured by Procollagen type I C-peptide ELISA kit (Takara Bio Inc.) according to the manufacturer’s instructions and plate readout was performed by measuring absorbance with a Wallac Victor2 1,420 multilabel counter (Perkin Elmer/Wallac) reader.

### Immunofluorescence staining and confocal microscopy/structure illumination microscope (SIM) imaging

Cells were fixed with 4% paraformaldehyde (Sigma-Aldrich) for 10 min and were then permeabilized and blocked in a blocking solution made of 0.1% (v/v) Triton-X (Sigma-Aldrich) and 10% (v/v) Fetal Bovine Serum (Gibco) diluted in DPBS (Gibco). The cells were then incubated overnight at 4°C with primary antibodies diluted in a blocking solution. Secondary antibody- and nuclear staining with DAPI (Thermo Fisher Scientific) was subsequently performed in the dark for 1 h at room temperature. The dilutions of the antibodies are specified in the Supplementary Table S3 section. The glass coverslips with the stained cells were then mounted on glass slides and the cells were imaged with an LSM700 (Zeiss, Germany) confocal microscope. Stained samples were stored at −20°C. Structure illumination microscope (SIM) imaging of scLSECs was performed with an ELYRA PS.1 microscope (Zeiss).

### Determination of autofluorescence and PDGFR-β by flow cytometry

Flow cytometry of scHSCs was performed at the Flow Cytometry Core Facility at Oslo University Hospital, Gaustad, on a BD FACSMelody™ Cell Sorter. scHSCs were fixed in 4% paraformaldehyde (Sigma-Aldrich) as a single-cell suspension before staining with an antibody for PDGFR-β (R&D Systems). Cells were washed in FACS buffer containing DBPS (Thermo Fisher Scientific) and 0.1% BSA (SEQENS). PDGFR-β was detected in the flow cytometer by an Alexa Fluor 488-A laser and autofluorescence was assessed using a Pacific Blue 405-540/50-A laser. Retinyl esters are excited at approximately 330 nm and optimal detection rates are hence obtained using a 350 nm UV laser (Vallverdú et al., 2021), which was not used in this study due to unavailability. A minimum of 10,000 cells were counted per sample. scHSC samples stained only with secondary antibody were used as negative controls for the detection of PDGFR-β and undifferentiated hESCs were used as the negative control for the autofluorescence. Analysis and data visualization was performed using the program Floreada (floreada.io).

### α-SMA quantification

α-SMA intensity of confocal images was quantified by an automated Python (03.09.2012) script, see Supplementary Method S1 for a schematic display of the workflow. The script is on GitHub: https://github.com/ingridwilhelmsen/a-SMA_analysis. Antibody dilutions and confocal microscope settings were equal for all samples that were compared in the image analysis to ensure that the input material was quantifiable.

### Vitamin A detection and quantification by UV image analysis

Vitamin A was indirectly measured by UV image analysis. scHSCs and pHSCs were imaged with an Axioscope fluorescence microscope (Zeiss) at a ×10 magnification under UV light and fields of 100% confluency were independently chosen. The UV images were acquired immediately after turning the UV light on and the microscope settings were equal for all images that were compared to ensure that the input material was quantifiable. Image analysis was performed in ImageJ (Java 8 32-bit) using two automated macros and the results were exported and used for statistical analysis. See Supplementary Method S2 for a schematic display of the workflow and the scripts of the automated macros.

### Senescence staining

scHSCs were either treated or not treated with DAPT (5 µM) during the long-term post-differentiation culture. The fraction of senescent cells was determined by Senescence Cells Histochemical Staining Kit (Sigma-Aldrich) according to the manufacturer’s instructions. The scHSCs were incubated overnight in the staining mixture of the kit. Stained cells were then imaged at ×10 magnification in a bright-field microscope and fields of 100% confluency were independently chosen. Image analysis was performed in ImageJ (Java 8 32-bit) using an automated macro (see Supplementary Method S3) and the results were exported and used for statistical analysis.

### Measurement of coagulation factor VIII secretion

Cell culture medium (at least 24 h old) was collected and stored at −80°C until the assay was performed. Analysis was performed with undiluted samples, thawed on ice, and analyzed with an FVIII quantification ELISA kit (Abcam), and plate readout was performed by measuring absorbance with a Wallac Victor2 1,420 multilabel counter (Perkin Elmer/Wallac) reader.

### Measurement of NO secretion

Nitric oxide (NO) was quantified using a Nitric oxide assay kit (Life Technologies) according to the manufacturer’s instructions. Due to the high antioxidant content of the cell culture medium, standards were prepared in the cell medium to equalize the possible negative effect of the antioxidants on the enzymatic reaction of the assay.

### *E. coli* bioparticle and AcLDL uptake assay

For the *E. coli* bioparticle uptake assay, a concentration of 163 opsonized *E. coli* (K-12 strain) BioParticles™, Alexa Fluor™ 594 conjugate (Thermo Fisher Scientific) was considered adequate. scLSECs were detached and cultured in a suspension of bioparticles in DMEM with 0.5% BSA for 1 h at 37 C°. Cells were then washed once in DPBS and fixed in a 4% PFA solution for 15 min. Cells were then washed three more times with PBS and stored at 4 C° until analysis.

For the AcLDL uptake assay, a suspension of 15 μg/mL Low-Density Lipoprotein from Human Plasma, Acetylated, Alexa Fluor™ 488 Conjugate (Alexa Fluor™ 488 AcLDL) (Thermo Fisher Scientific) was prepared in 0.5% BSA in DMEM. Cells were detached and incubated in the suspension for 20 min at 37 C°. Cells were then washed three times with PBS and stored at 4 C° until analysis.

Before flow cytometry, cells were blocked in a solution of 10% FBS in DPBS for 1 h at room temperature. Cells were then incubated with primary antibodies (anti-LYVE1, R&D, 1:60 dilution, and anti-VE-cadherin, Abcam, 1:500 dilution). Cells were then washed three times with PBS before incubation with secondary antibodies (see Materials) for 1 h at room temperature. After washing three more times with PBS, the stained cells were stored at 4 C° until flow cytometry analysis. HUVEC cells (Thermo Fisher Scientific) were used as the positive and negative controls.

Flow cytometry analysis was performed in biological triplicates with a BD Accuri C6 Plus Flow Cytometer and data was analyzed with the native software of the cytometer and histograms were made with Floreada (floreada.io) online software.

### Statistical analysis

Independent differentiations are treated as biological replicates (denoted “n”) throughout the study, while independent wells in a single differentiation are treated as technical replicates (denoted “N”).

**FIGURE 3:**
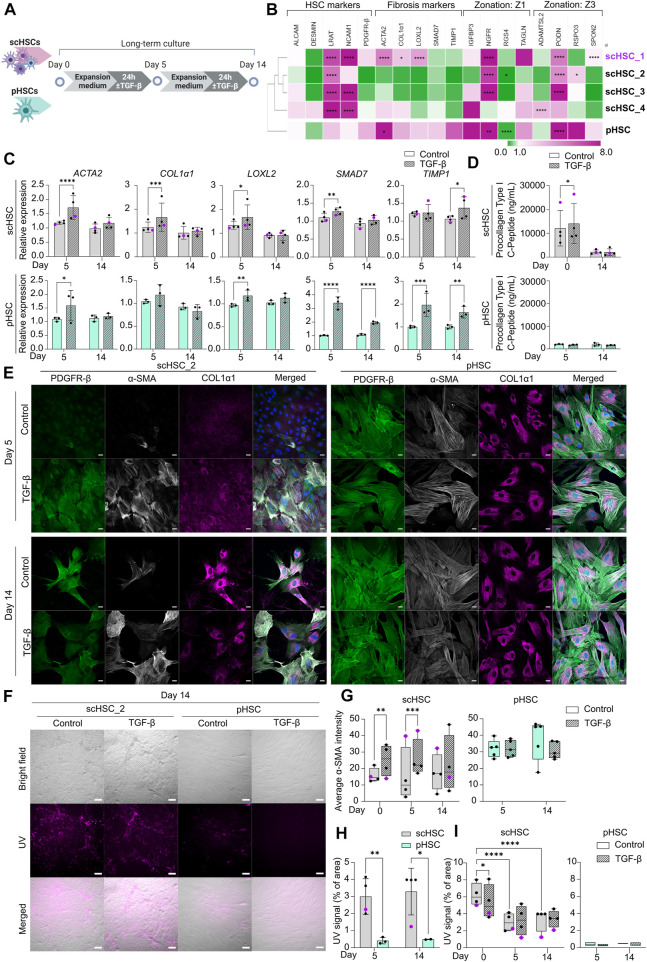
Characterization of Phenotypic and Functional Stability of scHSCs and pHSCs in Long-Term *in vitro* Culture. **(A)** Schematic representation of the long-term culture experiment of scHSCs and pHSCs. **(B)** Heatmap of the expression profile of scHSCs and pHSCs after 14 days in culture. Gene expression levels were normalized to day 0 of the culture for each respective cell line. The cell lines were clustered using the metric one minus Pearson correlation with complete linkage. Z1: Zone 1, Z3: Zone 3, N = 3 technical replicates. **(C)** Gene expression of fibrosis-associated markers in scHSCs and pHSCs during culture with or without treatment with TGF-β. Expression levels were normalized to the control. scHSCs: n = 4 independent differentiations, N = 3 technical replicates per differentiation. pHSCs: n = 1 donor, N = 3 technical replicates. **(D)** Secretion of procollagen type I C-Peptide by scHSCs and pHSCs either treated or not treated with TGF-β as measured by ELISA. scHSCs: n = 4 independent differentiations, N = 3 technical replicates per differentiation. pHSCs: n = 1 donor, N = 3 technical replicates. **(E)** Representative immunofluorescence confocal images of PDGFR-β, α-SMA, and COL1α1 in scHSC_2 and pHSCs during the culture. The cells were either treated or not treated with 25 ng/mL TGF-β for 24 h before fixation. Scale bars: 40 µm. **(F)** Representative bright-field- and UV images of scHSC_2 and pHSC after 14 days in culture with and without treatment with TGF-β. Scale bars: 100 µm. **(G)** Quantification of the average integral α-SMA intensity within PDGFR-β positive areas of immunofluorescence confocal images, comparing time points (day 0, 5, and 14 of the long-term culture), cell lines (scHSC_2 and pHSC) and treatments (control or treatment with TGF-β). scHSC: n = 4 independent differentiations, N ≥ 5 images of independent fields for each point. pHSC: N = 5 images of independent fields for each condition. **(H)** Vitamin A storage in scHSCs and pHSCs is indirectly measured by quantification of the UV signal. scHSCs: n = 4 independent differentiations, each point is the average of N = 3 technical replicates. pHSCs: n = 1 donor, N ≥ 2 images of independent fields. **(I)** Changes in the storage capacity of vitamin A of scHSCs and pHSCs at different time points in culture and after treatment with TGF-β. scHSC: n = 4 independent differentiations, N = 3 technical replicates for each point. pHSC: N ≥ 2 images of independent fields for each condition. Data from scHSC_1 (hESC-derived) is colored purple or shown as purple dots throughout the figure. See also Supplementary Figures S3, S4.

**FIGURE 4:**
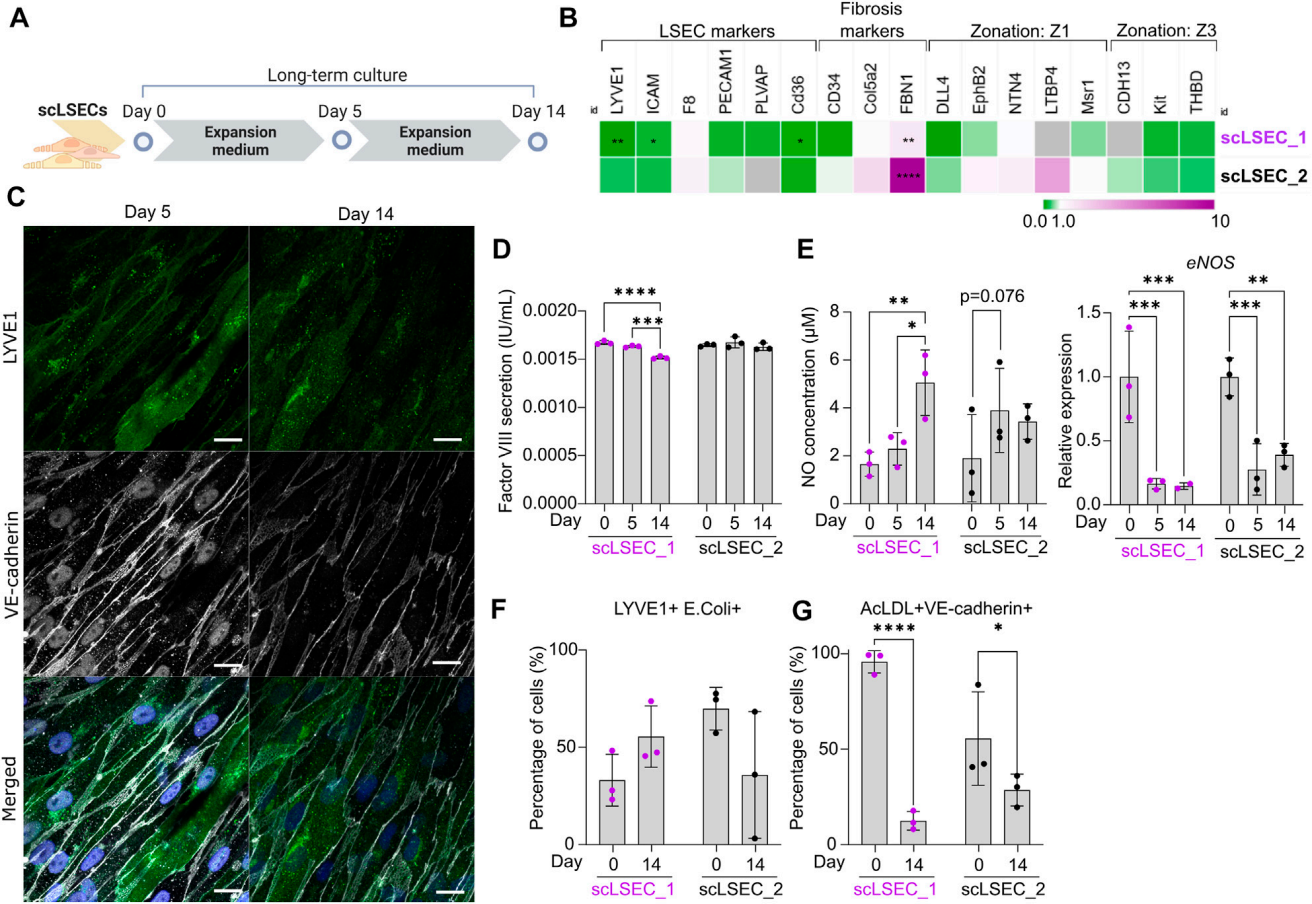
Characterization of Phenotypic and Functional Stability of scLSECs in Long-Term *in vitro* Culture. **(A)** Schematic representation of long-term culture of scLSECs. **(B)** Heatmap of the expression profile of scLSECs after 14 days in culture. The gene expression values were normalized to day 0 of the long-term culture for each respective cell line. Gray color indicates no detectable expression. Z1: Zone 1, Z3: Zone 3. N = 3 technical replicates. **(C)** Representative immunofluorescence confocal images of LYVE1 and VE-cadherin of scLSEC_2 on day 14 of the long-term culture. Scale bars: 20 µm. **(D)** Total coagulation factor VIII concentration in the cell culture medium of scLSECs on days 0, 5, and 14 as measured by ELISA. N = 3 technical replicates. **(E)** Total nitric oxide (NO) concentration in the cell culture medium and *eNOS* expression of scLSECs on days 0, 5, and 14 as measured by ELISA and qPCR respectively. *eNOS* expression values were normalized to day 0. N = 3 technical replicates. **(F)** Percentage of scLSECs on days 0 and 14 positive for LYVE1 and labeled *E. coli* bioparticles as measured by flow cytometry. N = 3 technical replicates. **(G)** Percentage of scLSECs on days 0 and 14 positive for VE-cadherin and labeled acetylated low-density lipoproteins (AcLDL) as measured by flow cytometry. N = 3 technical replicates. Data from scLSEC_1 (hESC-derived) are colored purple or shown as purple dots throughout the Figure. See also Supplementary Figure S5.

**FIGURE 5:**
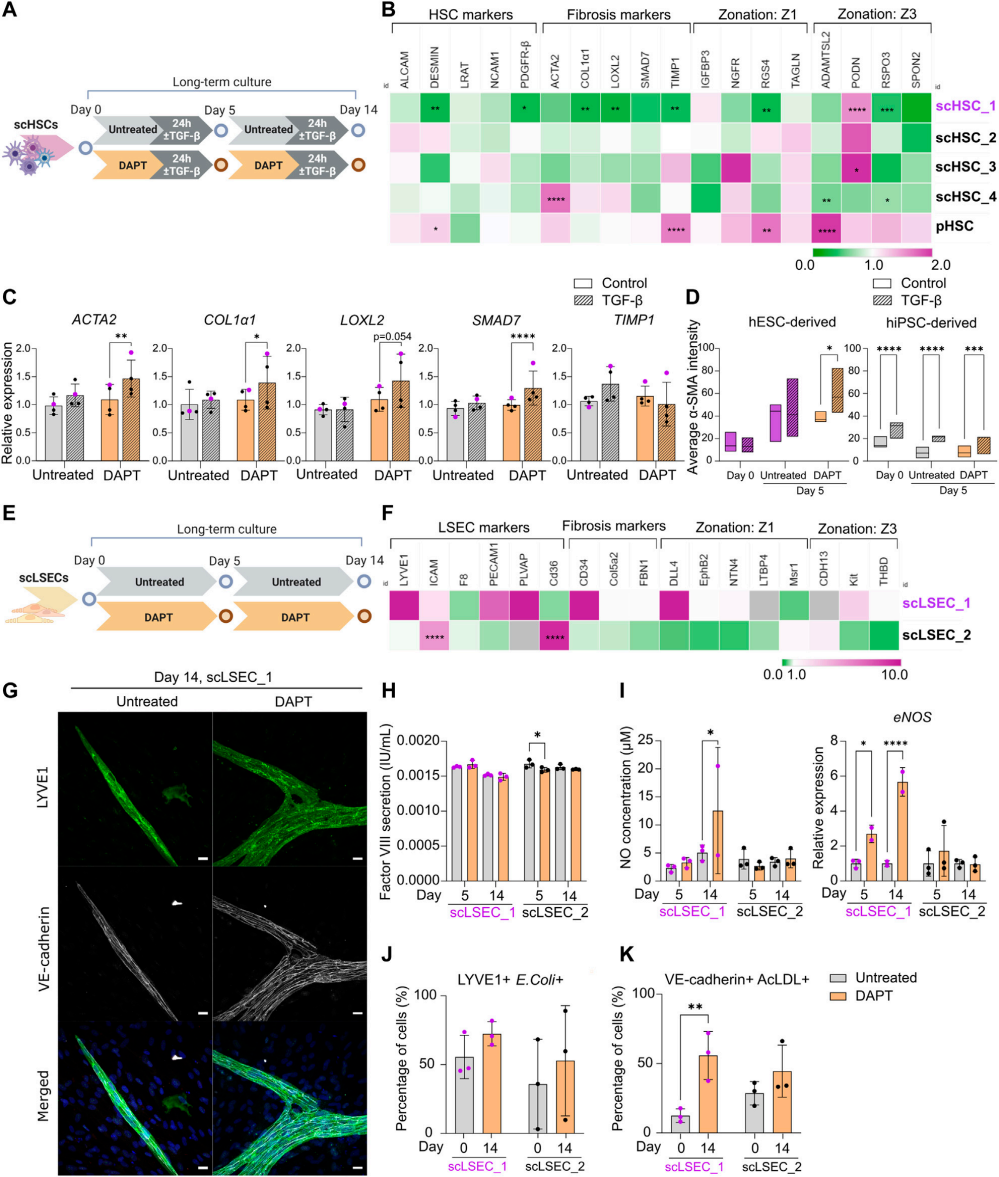
The Effect of Notch Pathway Inhibition on Gene Expression and Functionality of scHSCs and scLSECs in Long-Term *in vitro* Culture. **(A)** Schematic representation of the culture of scHSCs, comparing cells treated with DAPT and untreated cells. **(B)** Heatmap of the expression profile of scHSCs and pHSCs treated with DAPT relative to that of untreated cells after 14 days in culture. The gene expression levels of DAPT-treated samples on day 14 were normalized to the corresponding untreated samples on day 14. Z1: Zone 1, Z3: Zone 3. N = 3 technical replicates. **(C)** Gene expression of fibrosis-associated markers in untreated and DAPT-treated scHSCs on day 14 after treatment with TGF-β. Expression levels were normalized to the control for each condition. N = 4 independent differentiations, N = 3 technical replicates per differentiation. **(D)** Quantification of IF images measuring α-SMA intensity in untreated and DAPT-treated scHSCs on days 0 and 5. hESC-derived HSCs: n = 1 independent differentiation, N ≥ 5 images of independent fields, hiPSC-derived HSCs: n = 3 independent differentiations of three different hiPSC lines, N ≥ 5 images of independent fields for each cell line. **(E)** Schematic representation of the long-term culture of scLSECs, comparing cells treated with DAPT and untreated cells. **(F)** Heatmap of the expression profiles of scLSEC treated with DAPT relative to that of untreated cells after 14 days in culture. Gray color indicates no detectable expression. Z1: Zone 1, Z3: Zone 3. N = 3 technical replicates. **(G)** Representative immunofluorescence confocal images of LYVE1 and VE-cadherin of scLSEC_1 on day 14 of the long-term culture, comparing cells treated with DAPT and untreated cells. Scale bars: 20 µm. **(H)** Total coagulation factor VIII concentration in the cell culture medium of scLSECs on day 5 was measured by ELISA, comparing untreated and DAPT-treated samples. N = 3 technical replicates. **(I)** Total nitric oxide (NO) concentration in the cell culture medium and *eNOS* expression of scLSECs on days 0, 5, and 14 as measured by ELISA and qPCR respectively in untreated and DAPT-treated conditions. *eNOS* expression values were normalized to untreated samples for each day. N ≥ 2 technical replicates. **(J)** Percentage of scLSECs on days 0 and 14 positive for LYVE1 and labeled *E. coli* bioparticles as measured by flow cytometry, comparing untreated and DAPT-treated samples. N = 3 technical replicates. **(K)** Percentage of scLSECs on days 0 and 14 positive for VE-cadherin and labeled acetylated low-density lipoproteins (AcLDL) as measured by flow cytometry, comparing untreated and DAPT-treated samples. N = 3 technical replicates. Data from scHSC_1 and scLSEC_1 (hESC-derived) are colored purple or shown as purple dots throughout the figure. See also Supplementary Figures S6, S7.

**FIGURE 6:**
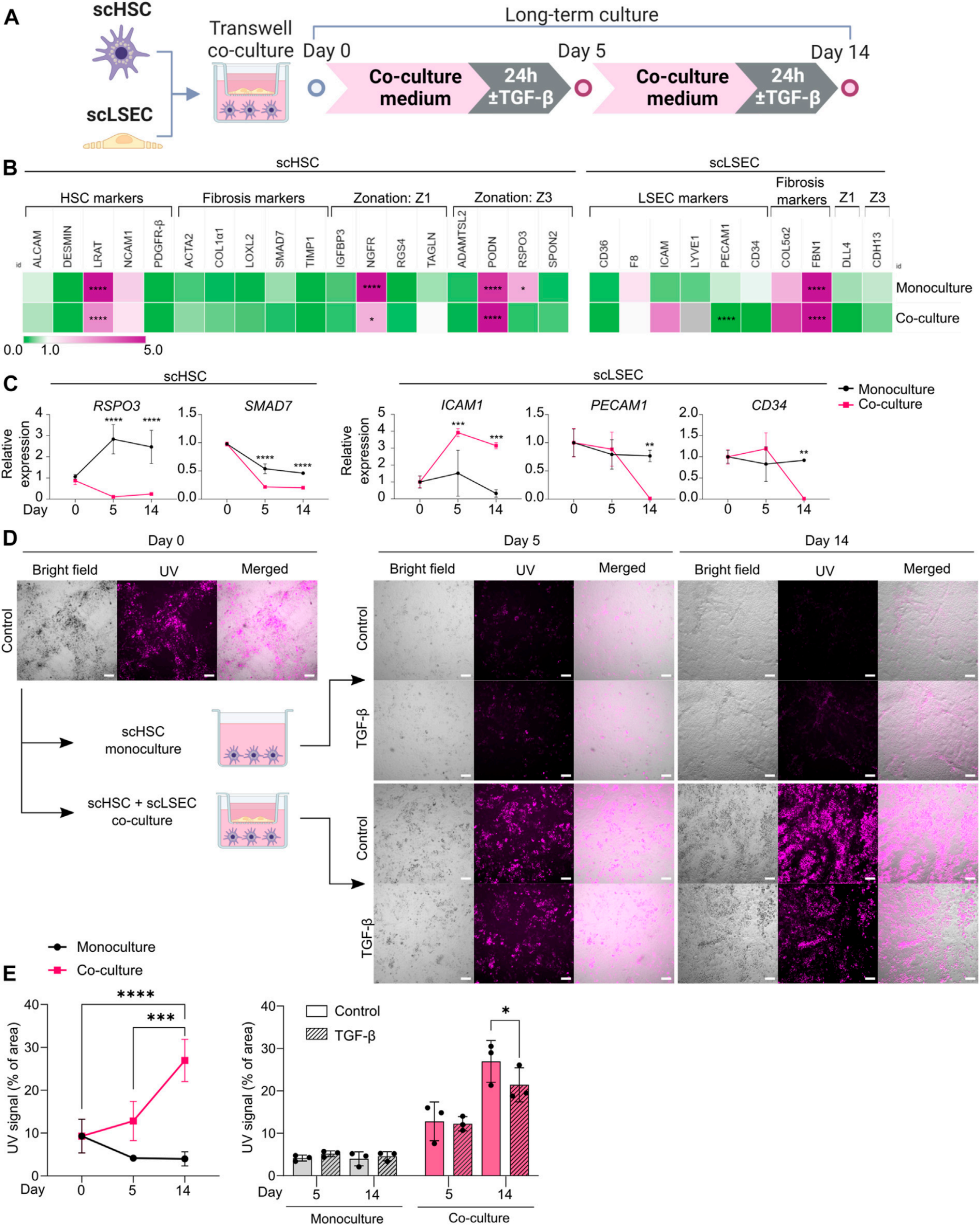
Comparison of Monoculture and Co-culture of scHSCs and scLSECs. **(A)** Schematic representation of scHSCs and scLSECs co-culture. **(B)** Heatmap of the expression profile of scHSCs and scLSECs grown in monoculture for 14 days. The gene expression level was normalized to day 0 of the culture for each respective cell line. Z1: Zone 1, Z3: Zone 3, gray color: Marker not detected. N = 3 technical replicates. **(C)** Gene expression of selected markers in scHSCs and scLSECs grown in monoculture or co-culture. The relative expression was normalized to day 0 for each respective cell line. N = 3 technical replicates. **(D)** Representative bright field- and UV images of scHSCs grown in monoculture and in co-culture with scLSECs with and without treatment with TGF-β. Scale bars: 100 µm. **(E)** Vitamin A storage in scHSCs grown in monoculture or co-culture with scLSECs for 14 days and the response to treatment with TGF-β. N = 3 technical replicates. See also Supplementary Figure S8.

Statistical analyses and significance were determined using the GraphPad Prism (9.3.1) software. When comparing two groups, a two-tailed, unpaired Mann-Whitney test was performed. When three or more groups were compared, a two-way ANOVA, uncorrected Fisher’s least significant difference (LSD) was performed. Culture conditions and scHSC/scLSEC lines were used as variables in the ANOVA analyses. ANOVA analyses performed for the heatmaps were performed with Šídák’s multiple comparisons correction (Figures 1C, 2C, 3B, 4B, 5B,F, 6B).

Results were considered statistically significant at *p* < 0.05 with the following markings: *p < 0.05, **p < 0.005, ***p < 0.0005, ****p < 0.0001. Results presented as bar graphs are expressed as the mean ± standard deviation (SD). Box and whisker plots display the five-number summary of the data, representing the minimum and maximum values at the whiskers, the first and third quartiles (Q1 and Q3) of the data within the box, and the median (Q2) at the line in the box. Boxplots without whiskers include all the data within the box and the median at the line in the box.

### Figure creation

Schematic figures were created with BioRender (BioRender.com). Heatmaps were created with Morpheus (https://software.broadinstitute.org/morpheus). Graphs were created with GraphPad Prism (9.3.1).

### Ethics

hESC and hiPSC work is under the following ethical approval: REK approval #50786: “The development of “organ-on-a-chip” systems for the *in vitro* study of physiological responses of organs, organ-like systems in metabolically active tissues at the center of excellence—Hybrid technology, IMB, UiO”.

## Results

### Differentiation and characterization of pluripotent stem cell-derived hepatic stellate cells and liver sinusoidal endothelial cells

Protocols for differentiating scHSCs and scLSECs have recently been established (Coll et al., 2018; Gage et al., 2020; Vallverdú et al., 2021). In this study, modified versions of the protocols for the production of scHSC and scLSEC lines were established using one hESC line (H1: sc_1) and three hiPSC lines (WTC-11: sc_2, WTSli28-A: sc_3, and WTSli013-A: sc_4). Due to PSC line-dependent differences in the differentiation efficiency of the protocols, all four PSC lines were used for the production of scHSCs while two PSC lines, sc_1 and sc_2, were used for the production of scLSECs.

Figure 1A illustrates the 12-day scHSC differentiation protocol. Real-time quantitative polymerase chain reaction (RT-qPCR) analysis revealed decreased expression of the pluripotency marker octamer-binding transcription factor 4 (*OCT4*) (Figure 1B). Markers that are expressed in developing and mature HSCs were upregulated throughout the differentiation protocol; platelet-derived growth factor β (*PDGFR- β*), a surface marker of HSCs (Henderson et al., 2013), was universally upregulated, and HSC-typical intermediate filament *desmin* increased in all cell lines except scHSC_4 (Puche et al., 2013). Moreover, we detected an increase in the expression of the activated leukocyte cell adhesion molecule (*ALCAM*) (Asahina et al., 2009) and the cell surface marker neural adhesion molecule (*NCAM1*) (Knittel et al., 1996; Asahina et al., 2009). The expression pattern of lecithin retinol acyltransferase (*LRAT*), an enzyme required for all retinyl ester synthesis (Blaner et al., 2009a), was consistent with published work on scHSC differentiation (Coll et al., 2018). To benchmark the differentiated scHSCs to commercially available pHSCs, the transcriptional levels of HSC markers were compared (Figure 1C). *ALCAM* and *PDGFR-β* were expressed at levels similar to that of pHSCs with minor variations. Interestingly, *LRAT* was detected at significantly higher levels in scHSCs. The loss of vitamin A-storing lipid droplets is a hallmark of HSC activation upon liver injury (Tsuchida and Friedman, 2017) and although the role of LRAT in HSC activation is unclear, LRAT has been suggested as a marker for HSC (Nagatsuma et al., 2009). The HSC markers actin alpha 2 (*ACTA2*) and collagen type 1 alpha 1 (*COL1α1*), associated with an activated phenotype (Kitto and Henderson, 2021), showed a tendency to be expressed at either a lower or equal level in the scHSCs.

Both bright-field- (Supplementary Figure S1A) and confocal imaging revealed that pHSCs were notably larger in appearance compared to scHSCs. The transition from smaller to larger and more elongated cells has previously been characterized as a hallmark of human HSC activation (El Taghdouini et al., 2015). The presence of PDGFR-β and NCAM1 complemented by the cytoskeletal HSC marker vimentin (Zhang et al., 2018) was confirmed by immunostaining and confocal imaging (Figure 1D and Supplementary Figure S1B). Quiescent HSCs store vitamin A intracellular lipid droplets in the form of retinyl esters, which are excited at approximately 330 nm (Blaner et al., 2009a). Vitamin A was identified in the scHSCs by autofluorescence in flow cytometry analysis, revealing right-shifting of the population as a result of autofluorescence in both scHSC_1 and scHSC_2 (Figure 1E). Imaging under UV light confirmed that all scHSC lines produced autofluorescent signals, while no autofluorescence was detected in the pHSCs (Supplementary Figure S1E). Hence, after the 12-day differentiation protocol, we observed an overall favorable phenotype in scHSCs compared to commercially available pHSCs.

As a functionality test for the scHSCs, we chose the ability to respond to a fibrosis-inducing stimulus by TGF-β. Activation of the TGF-β/Smad signaling pathway is well-characterized as a core pathway of fibrosis (Distler et al., 2019) and HSCs are potently activated by elevated concentrations of TGF-β (Dewidar et al, 2019). Data from all scHSC lines revealed transcriptional elevation of the fibrosis-related markers *ACTA2* and *COL1α1* as well as the TGF-β/Smad signaling pathway target genes lysyl oxidase-like 2 (*LOXL2*), SMAD family member 7 (*SMAD7*) and TIMP metallopeptidase inhibitor 1 (*TIMP1*) after both 24 and 48 h of treatment with TGF-β (Figure 1F; scHSC_1 visualized in purple). pHSCs exhibited similar patterns with a notable lack of *ACTA2* induction upon activation. Subsequently, the presence of PDGFR-β, both an HSC marker and a receptor in a fibrosis-implicated pathway (Distler et al., 2019), alpha smooth-muscle actin (α-SMA), and collagen type I alpha 1 chain (COL1α1) was determined by IF (Figure 1G and Supplementary Figure S1D; α-SMA quantified in Figure 1H). Increased intensity of all the IF-marked proteins was observable in the images of scHSC_2 treated with TGF-β (Figure 1G, see also Supplementary Figure S1D), indicating an induction of an activated-like phenotype in response to the pro-fibrotic stimulus. Quantification of the intensity of the α-SMA IF signal (see Supplementary Methods S1 and script: https://github.com/ingridwilhelmsen/a-SMA_analysis) revealed a significant increase in α-SMA intensity in all hiPSC-derived scHSC upon treatment with TGF-β, an effect that was not reproduced in the hESC-derived scHSC_1 or pHSCs. Lastly, the impact of the pro-fibrotic stimulus on the ability of the scHSCs to store vitamin A was assessed by quantifying UV positivity, whereby scHSC_2 exhibited a significant reduction in UV signal after treatment with TGF-β (Supplementary Figure S1F).

Collectively, the results indicate that the differentiation protocol produced scHSCs that exhibited properties of a quiescent HSC phenotype with an ability to respond to a pro-fibrotic stimulus induced by TGF-β. However, we did note a differential response between cell lines in the α-SMA IF and in the UV signal, the latter indicative of vitamin A storage.

scLSECs were differentiated following a modified version of a published protocol (Figure 2A) (Gage et al., 2020). During the differentiation, an overall loss of the expression of *OCT4* was seen in the two tested cell lines (Figure 2B). The upregulation of the endothelial cell markers platelet endothelial adhesion molecule 1 (*PECAM1*) (Woodfin et al., 2007), as well as hematopoietic progenitor cell antigen *CD34 (CD34)* (Vestweber, 2008) and lymphatic vessel endothelial hyaluronan receptor 1 *(LYVE1)* (Helle et al., 2015), was evident at the expansion stage of the differentiation. Specification toward scLSEC was accompanied by stable or downregulated expression of *PECAM1* and *CD34* (Gage et al., 2020). Next, we benchmarked the scLSEC_2 line to commercially available primary human liver sinusoidal endothelial cells (pLSEC, Figure 2C) and human hepatic endothelial cells (pHLEC, Figure 2D). Comparison to pLSEC revealed differences between the cell lines; increased expression of LSEC markers *ICAM* and *CD36* was observed in scLSEC_1 while *LYVE1* and *PECAM1* were increased in scLSEC_2. None of the tested LSEC markers exhibited a significantly lower expression in the scLSECs. Notably, *LYVE1* expression was increased by approximately 52-fold in scLSEC_2 compared to pHLECs, demonstrating a specific LSEC-like identity in the scLSECs rather than a general hepatic endothelial cells-like identity as presumably present in the pHLECs. Nevertheless, we observed comparable expression levels of coagulation factor VIII encoding gene (*F8*) in the scLSECs and pHLECs.

The morphology of differentiated scLSECs varied between cells derived from hiPSCs and hESCs. High-magnification bright-field images revealed that scLSEC_1 derived from hESCs showed a polygonal shape with frequent discontinuations. In contrast, scLSEC_2 derived from hiPSCs showed a fusiform shape with more frequent and smaller circular surface structures. scLSEC_2 more closely resembled the morphology of the pLSECs (Supplementary Figure S2A). Collectively, these observations suggest that, while both hESC- and hiPSC-derived scLSECs show signs of maturity, there is a PSC line-dependent diversity in scLSEC.

Confocal imaging of scLSEC_2 labeled for LYVE1 and VE-cadherin showed the expression of the markers in the majority of the cell populations, similar to the expression observed in pLSECs (Figure 2E). LYVE1 is a surface-scavenging receptor and VE-cadherin is a common adherent-junction protein present in all vascular endothelial cells (Vestweber, 2008). Notably, VE-cadherin was spatially distributed in ring-like structures in the cell-cell junction areas. Lastly, scLSEC_2 samples were labeled for LYVE1 and non-phosphorylated β-catenin (non-P-β-catenin). The samples, which were imaged with a structure illumination microscope (SIM), illustrated the expression of non-P-β-catenin in a repeated ring-like motif (Figure 2F). LYVE1 is seen to be present independent of the non-P-β-catenin structures. The ring-like elements had a diameter of around 1–3 µm. This diameter corresponds with that of sieve plates, which are clustered groups of fenestrae, in LSECs (Helle et al., 2015). However, the data does not allow conclusive identification of such structures.

Next, the functionality of the scLSECs was assessed by their ability to secrete coagulation factor VIII and nitric oxide (NO). The production of factor VIII, vital for a functioning coagulation system, is a task of LSECs while NO is a small signaling molecule essential to preserve HSC quiescence that is produced by LSECs in small amounts in the homeostatic liver (Iwakiri and Kim, 2015). Enzyme-linked immunosorbent assay (ELISA) revealed that both scLSEC lines produced and secreted factor VIII in the culture media (0.006–0.008 IU/mL) (Figure 2G). Contrastingly, no factor VIII was detected within the solid detection range of the assay in samples from pLSEC after 1 and 5 days of culturing (data not shown). NO production and secretion were detected in both scLSEC lines within the range of approximately 1.5 and 2.0 µM (Figure 2H).

The scavenging function of the scLSECs was tested by the endocytic uptake of fluorochrome-labeled acetylated low-density lipoprotein (AcLDL) and bacterial bioparticles using flow cytometry analysis. Figures 2I, J show that fractions of both scLSEC lines were double positive for either LYVE1 and *E. coli* bioparticles (33.1% of scLSEC_1% and 69.8% of scLSEC_2) or VE-cadherin and AcLDL (95.9% of scLSEC_1% and 55.6% of scLSEC_2).

Overall, the results underline the robustness of the differentiation protocol, leading to scLSECs with LSEC-like phenotypes and detectable physiological functionality. While the results suggest that PSC-derived scLSECs constituted heterogeneous cell populations, they exhibited significant cell type-specific secretory and endocytic functions.

### Long-term culture of pluripotent stem cell-derived hepatic stellate cells and liver sinusoidal endothelial cells

After differentiation, PSC-derived cells should show phenotypical and functional stability to enable reliable and robust disease modeling. Hence, scHSCs, and scLSECs were cultured in their respective expansion media (see Supplementary Table S1 for media compositions) for 14 days, and the impact of both time in culture and PSC line on the cell-specific phenotype and functionality were characterized. As shown in the scheme of Figures 3A, 4A, scHSCs and scLSECs were assessed before (day 0), during (day 5), and at the end (day 14) of the culture. Data from the hESC-derived scHSC_1 and scLSEC_1 is colored purple in all the figures.

scHSCs differentiated from four PSC lines and pHSCs from one donor were examined (Figure 3A). Figure 3B shows the relative changes in the expression of selected genes on day 14 of the culture compared to day 0 of each respective cell line measured by RT-qPCR and sub-divided into 4 groups: HSC markers, fibrosis markers, zone 1 markers, and zone 3 markers (see also Supplementary Figure S3A). The analysis revealed significant, cell line-dependent effects and differences*.* The HSC markers *LRAT* and *NCAM1* were upregulated in all cell lines. The time-dependent increase of *NCAM1* expression is associated with activation and myofibroblast formation (Rosenberg et al., 2011; Hintermann and Christen, 2019). Interestingly, however, fibrosis-related markers displayed a trend of downregulation (scHSC_2, scHSC_3) or only slight elevation (scHSC_4) in hiPSC-derived scHSCs. On the contrary, several fibrosis markers (*ACTA2*, *COL1α1,* and *LOXL2*) were elevated in the hESC-derived scHSC_1. The scHSC_1 line was thus similar to pHSCs, which exhibited a modest elevation of all fibrosis markers. Except for notable upregulations of the nerve growth factor receptor (*NGFR*) (Dobie et al., 2019) and podocan (*PODN*) (Hutter et al., 2013), no discernible pattern in the expression of zonation-related genes was observed.

Next, we tested the impact of the long-term culture on the functionality of scHSCs and pHSCs. RT-qPCR analysis of fibrosis-related genes showed that overall, scHSCs and pHSCs retained the capability to respond to a pro-fibrotic stimulus induced by TGF-β after 5 days of culturing (Figure 3C). However, this ability had been lost at day 14 of the culture except for *TIMP1*. ELISA for procollagen type I C-peptide revealed increased concentrations of the protein in the culture media from scHSCs treated with pro-fibrotic stimulus on day 0 but not on day 14 (Figure 3D). In contrast, pHSCs displayed a relatively low level of collagen secretion with no observable increase upon treatment with TGF-β.

The morphology of scHSCs and pHSCs as well as protein production and distribution of PDGFR-β, α-SMA, and COL1α1 were assayed during the culture (Figure 3E and Supplementary Figure S4D; α-SMA quantified in Figure 3G). We noted differences in phenotype and functionality between hESC-derived scHSC_1, hiPSC-derived scHSCs, and pHSCs. Notably, pHSCs consistently displayed a more activated phenotype compared to hiPSC-derived scHSCs during the culture, with pHSC being larger with a pronounced presence of α-SMA and COL1α1. The morphology of the hESC-derived scHSC_1 more closely resembled the morphology of pHSC on both days 5 and 14 (Supplementary Figure S4D). Upon pro-fibrotic stimulation, sub-populations of cells with an activated phenotype were induced in all hiPSC-derived scHSC lines on days 5 and 14. hESC-derived scHSC_1 and pHSC, in contrast, did not show an increased activated state with the same treatment. Quantification of the α-SMA IF signal intensity in scHSCs confirmed an intensity increase after a pro-fibrotic stimulus at days 0 and 5, which declined at day 14. In pHSCs, no change was detected in the α-SMA IF signal.

Lastly, vitamin A storage was measured by imaging under UV light (Figure 3F), as the release of vitamin A is a characteristic of HSC activation (Tsuchida and Friedman, 2017). Figure 3H shows that scHSCs produced a higher UV signal compared to pHSC which exhibited a signal of negligible intensity (covering less than 0.5% of the image area). However, as shown in Figure 3I, the signal in scHSCs significantly decreased after both 5 and 14 days in culture compared to day 0. Moreover, the UV signal was unaffected by a pro-fibrotic treatment in both scHSC and pHSC on days 5 and 14. Noteworthy, hESC-derived scHSC_1 were at the lower end of the UV signal range in the scHSC cluster, and hence closer to pHSCs.

Similar to pHSCs, scHSCs are impacted by time in culture as they gradually lose their capacity to respond to the pro-fibrotic stimulus induced by TGF-β when cultured for up to 14 days. Noteworthy, hESC-derived scHSC_1 acquired a phenotype more closely resembling that of pHSCs, with characteristics typical for activated HSCs. Particularly hiPSC-derived scHSCs can be considered a good alternative to pHSCs as they retain comparatively more quiescent-like characteristics and a stronger response to pro-fibrotic treatment.

Next, scLSECs differentiated from hESCs (scLSEC_1) or hiPSCs (scLSEC_2) were cultured for 14 days as illustrated in Figure 4A. As the commercially available pLSECs rapidly lose LSEC-like characteristics after short-term *in vitro* cultivation (Elvevold et al., 2008), the long-term experiments were performed without benchmarking to primary cells. The gene expression of common LSEC-, pro-fibrotic- and zonation-specific markers was analyzed by RT-qPCR, examining the relative change in expression on day 14 of the culture compared to day 0 (Figure 4B, see also Supplementary Figure S5A). In both cell lines, we observed a trend of downregulation of LSEC-specific gene expression over time. In contrast, the fibrosis-related marker *FBN1* was significantly upregulated in both scLSEC lines. Like scHSCs, no discernible pattern was observed in the LSEC zonation markers. Next, the morphology of scLSECs was observed by bright-field microscopy on day 14 (Supplementary Figure S5B). scLSECs displayed a less endothelial-like morphology compared to day 0 as evidenced by a reduction in LSEC-typical fusiform cell bodies and an increase in fibroblast-like elongations.

Imaging of IF-stained scLSEC_2 visualized the preserved presence of LYVE1 and VE-cadherin (Figure 4C). Contrastingly, scLSEC_1 displayed a much weaker signal of both LYVE1 and VE-cadherin (Supplementary Figure S5C). The decline in observable LYVE1 and VE-cadherin co-expression in the scLSEC_1 line suggests the cells lose LSEC-specific characteristics with time in culture.

The functionality of scLSECs was tested during the culture by the same assays as earlier described. Factor VIII secretion by both scLSEC lines remained stable during the culture with a small decrease detected in the scLSEC_1 line (Figure 4D). In the liver, NO is synthesized by the nitric oxide synthases (NOS) endothelial NOS (eNOS), and inducible NOS (iNOS). eNOS expression is constitutive in LSECs and small amounts of eNOS-derived NO maintain liver homeostasis while inhibiting pathogenesis. Contrastingly, iNOS-derived NO is induced in pathological conditions and results in high NO concentrations which act as damaging reactive nitrogen species (Iwakiri and Kim, 2015). Both scLSEC lines displayed trends of increased NO secretion during the culture, however, accompanied by decreased e*NOS* expression (Figure 4E). These results suggest that the increase in NO release was not eNOS-related, and can therefore be considered as a sign of cellular stress in the scLSEC culture.

Flow cytometry analysis detected no statistically relevant changes in the fractions of scLSEC_1 and scLSEC_2 double positive for LYVE1 and *E. coli* bioparticles after 14 days in culture (Figure 4F). In contrast, the fraction of the scLSEC_1 cells double positive for VE-cadherin and AcLDL was greatly reduced after 14 days in culture, with a significant decrease from 95.9% on day 0 to 12.5% on day 14 (Figure 4G). The scLSEC_2 line demonstrated the same trend.

Overall, scLSEC exhibited a decline in their cell type-specific expression profile and phenotypic attributes over time in culture, as well as in the functional parameters of NO secretion and AcLDL uptake. We note a differential response between the hESC-derived scLSEC_1 and the hiPSC-derived scLSEC_2, as scLSEC_1 exhibited reduced expression of LSEC-specific markers.

### The effect of Notch inhibition on long-term culture of pluripotent stem cell-derived hepatic stellate cells and liver sinusoidal endothelial cells

As a possible approach to preserve phenotype and functionality over time, we investigated the impact of Notch signaling inhibition on PSC-derived scHSCs and scLSECs. DAPT (γ-secretase inhibitor) was chosen as it has been tested for the treatment of liver fibrosis in rat models (Chen et al., 2012; Wang et al., 2017; Fang et al., 2022). Both HSCs and LSECs were cultured in the presence of 10 uM DAPT for 14 days of culture (Figures 5A,E). Successful targeting of the Notch pathway was confirmed by RT-qPCR analysis revealing transcriptional downregulation of Notch pathway markers in scHSCs (*JAG1* and *HEY1*) and scLSECs (*HEY1* and *HES1*) (Supplementary Figure S6A).

Changes in the expression profile of DAPT-treated scHSCs compared to untreated scHSCs on day 14 were measured by RT-qPCR (Figure 5B). Notably, the hESC-derived scHSC_1 line - which was the PSC line that displayed the most activated phenotype in the long-term culture (Figure 3) - showed the most significant response to DAPT treatment. This was evident in the downregulation of HSC-markers *desmin* and *PDGFR-β* (both associated with fibrotic development) as well as the fibrosis-related markers *COL1α1*, *LOXL2,* and *TIMP1* (Ying et al., 2017; Zhang et al., 2018). Overall, DAPT-treated scHSCs displayed an increase in sensitivity to a pro-fibrotic stimulus on day 14 on the level of gene expression as evident in the elevation of fibrosis-related markers *ACTA2*, *COL1α1*, *LOXL2,* and *SMAD7*, which was especially prevalent in the scHSC_1 line (Figure 5C, scHSC_1 data visualized in purple). Quantification of α-SMA IF intensity also indicated that scHSC_1 was more sensitive to the pro-fibrotic stimulus after treatment with DAPT (Figure 5D, Supplementary Figure S7A), whereas hiPSC-derived scHSCs were sensitive to TGF-β both in the presence or absence of DAPT (Figure 5D). A beneficial effect of DAPT treatment was not observed in pHSCs (Supplementary Figure S6B).

Next, the expression of DAPT-treated scLSECs was compared to untreated scLSECs on day 14 (Figure 5E). No clear trends in the expression of the tested genes were detected in either cell line despite a differential response of scLSEC_1 compared to scLSEC_2 (Figure 5F). However, imaging of scLSECs cultured in DAPT-supplemented media revealed a change in morphology as scLSEC_1 formed LYVE1-and VE-cadherin positive branch-like condensations on top of the otherwise LYVE1-and VE-cadherin negative monolayer of cells (IF images in Figure 5G; bright-field in Supplementary Figure S6E). The same effect was observed to a lesser extent in scLSEC_2 which expressed LYVE1 and VE-cadherin more consistently (Supplementary Figure S7B).

LSEC functionality assays detected stable concentrations of factor VIII and NO in the presence and absence of DAPT during the culture except for a minor decrease in factor VIII for scLSEC_2 on day 5 and a possible increase in NO for scLSEC_1 on day 14 (Figures 5H, I). Notably, *eNOS* expression was elevated in the presence of DAPT for scLSEC_1, which is an indicator of LSECs under homeostatic conditions (Iwakiri and Kim, 2015). This is consistent with existing literature that demonstrates decreased LSEC *eNOS* expression upon Notch activation (Duan et al., 2018). Similarly, the fraction of cells double positive for LYVE1 and *E. coli* bioparticles displayed a positive trend in the presence of DAPT in both scLSEC lines (Figure 5J). Lastly, the average proportion of scLSEC_1 positive for VE-cadherin and labeled AcLDL was significantly increased from 12.5% to 55.9% after treatment with DAPT, and a positive trend was observed in scLSEC_2 (Figure 5K). These results suggest that DAPT can have a beneficial effect in preserving the NO secretion and scavenging capacity of hESC-derived scLSEC_1.

Collectively, the data suggest that Notch inhibition by DAPT can positively affect aspects of functionality and phenotype scHSCs and scLSECs during long-term culture in a cell line-dependent manner. The beneficial effect of DAPT treatment was more visible in hESC-derived scHSCs and scLSECs which were more negatively impacted by prolonged culture, when compared to their hiPSC-derived counterparts.

### Long-term Co-Culture of scHSCs and scLSECs for the Assessment of phenotypical and functional changes

In the hepatic sinusoids, HSCs and LSECs form a functional unit. To investigate whether scHSCs and scLSECs influenced each other in long-term culture, the cells were co-cultured in transwell plates for 14 days (Figure 6A) whereby scHSC_2 and scLSEC_2 were chosen due to their favorable quiescent-like phenotypes (Figures 3, 4).

Figure 6B shows RT-qPCR gene expression data on day 14 relative to day 0, comparing scHSCs and scLSECs grown in mono- and co-culture (see also Supplementary Figure S8A). For both scHSCs and scLSECs, the co-culture had an overall minimal impact on gene expression except for the genes presented in Figure 6C. However, notable alterations in the co-cultured scHSCs included the Wnt-signalling agonist R-spondin 3 (*RSPO3*) which was significantly upregulated in the monoculture but stayed at a low level in the co-culture. *RSPO3* overexpression has been reported in activated HSCs and fibrotic liver tissue (Yin et al., 2016). The fibrosis-related marker *SMAD7* showed a lower expression in co-cultured scHSCs. This indicates that co-culture with scLSECs can positively affect scHSCs. Similarly, scLSECs displayed favorable features in the co-culture (Figure 6C). The trend of increased expression of the LSEC marker *ICAM1* and the significant decrease of *PECAM1* is indicative of an LSEC-specific phenotype. *PECAM1* is known to be highly expressed on vascular endothelial cells but downregulated in healthy LSEC (Poisson et al., 2017). Furthermore, in scLSECs, the fibrosis-related marker *CD34* displayed downregulation in the co-culture.

The most notable effect of the co-culture was observed in the increased capacity for vitamin A storage in scHSCs as measured by UV autofluorescence. Images taken under UV light demonstrated a large increase in the fluorescent signal of scHSCs grown in co-culture (Figure 6D; UV signal quantified in 6E). Furthermore, the functionality of the scHSCs in the co-culture was assessed by their reactivity to a pro-fibrotic stimulus by TGF-β. Quantification of the UV signal revealed a statistically significant decrease upon exposure to TGF-β in co-culture that was absent in monoculture (Figure 6E). As release of vitamin A is a hallmark of HSC activation, the results may suggest a functional increase of the co-cultured scHSCs.

Collectively, the long-term co-culture of scHSCs and scLSECs shows evidence of an improved quiescent state in both scHSCs and scLSECs, and an enhanced functional response to a pro-fibrotic stimulus in scHSCs.

## Discussion

The time-dependent decline in cell-specific phenotypic traits and functionality is a well-known limitation of *in vitro* cell culture models (Sancho-Bru et al., 2005; Xie et al., 2013; Sakai and Yoshimura, 2021). It is, therefore, crucial to thoroughly analyze the stability of cell types if they are to be included in *vitro* disease platforms. Our work is the first systematic study of phenotypic and functional changes of hESC- and hiPSC-derived scHSCs and scLSECs during prolonged *in vitro* culture, assessing the applicability of PSC-derived cells in liver disease modeling.

Our study illustrates the importance of time as a variable in experiments using PSC-derived scHSCs and scLSECs. We discovered that scHSCs partly retain functional characteristics associated with a quiescent HSC phenotype after 5 days in culture. However, the activation capacity of the scHSCs was lost on day 14 of the culture. These results suggest that PSC-derived scHSCs are affected by *in vitro* culture similarly to pHSCs, which are known to acquire an activated phenotype in 2D cultures (Sancho-Bru et al., 2005; Sakai and Yoshimura, 2021). On the other hand, our data suggest that scHSCs may retain a more quiescent-like phenotype than pHSCs, evident by the comparably low expression of fibrosis-related genetic markers and high storage capacity of vitamin A. This establishes PSC-derived scHSCs as an attractive alternative to pHSCs for *in vitro* modeling. Similarly, most of the LSEC-specific characteristics were altered in scLSEC after 14 days in culture, including a reduction of LSEC surface markers LYVE1 and VE-cadherin and a lowered capacity for endocytosis. Intrinsic identity markers of LSEC were downregulated at the end of the culture and the common fibrosis marker *FBN1* was upregulated, indicative of a deteriorating LSEC identity.

We also describe cell line-dependent differences between the hESC- and hiPSC-derived scHSCs and scLSECs. scHSCs derived from hESCs (scHSC_1) exhibited a phenotype more closely resembling that of the pHSCs during the culture compared to hiPSC-derived scHSCs (scHSC_2, scHSC_3, and scHSC_4). A similar difference between the cell lines was also seen in scLSECs, whereby hESC-derived scLSEC_1 tended to decline more significantly, exemplified by gene expression data and reduced endocytic capability. These results highlight key differences between hESC- and hiPSC-derived scHSCs and scLSECs.

Notch activation is implicated in the damage of liver homeostasis and LSEC dedifferentiation (Duan et al., 2018), and the Notch inhibitor DAPT has been proposed as a therapeutic agent for the treatment of liver fibrosis (Chen et al., 2012; Wang et al., 2017; Fang et al., 2022). DAPT was therefore tested as a potentially beneficial medium supplement for HSC and LSEC quiescence. Consistent with existing literature, our results show a cell line-dependent modestly positive effect of DAPT treatment, displaying a greater phenotypic and functional improvement in both hESC-derived scHSC_1 and scLSEC_1 compared to their hiPSC-derived counterparts. This is notable as hESC-derived cells exhibit a more activated and hence less healthy phenotype in long-term culture. We detected no negative effects of DAPT on the tested cells. Consequently, Notch inhibitors may be suitable as components in an optimized culture medium.

A limitation of monoculture-based models can be an exceedingly simplistic recapitulation of physiology and pathology. In our study, we present a co-culture of scHSCs and scLSECs. scHSCs benefitted from the co-culture by showing an increase in vitamin A storage, which is a characteristic of a quiescent HSC phenotype (Tsuchida and Friedman, 2017). scLSECs also benefited from the co-culture as evidenced by favorable changes in LSEC- and fibrosis-related markers.

In summary, we conducted a systematic study to assess the long-term culture of PSC-derived scHSCs and scLSECs, highlighting the importance of time-in-culture as a variable of *in vitro* models. Additionally, we describe PSC line-dependent phenotypical and functional variability of scHSCs and scLSECs, whereby hESC-derived cells were more susceptible to time-dependent decline in cell-specific traits. Notch inhibition by DAPT displayed a modest but relevant improvement of cell performance most prominent in hESC-derived scHSCs and scLSECs and can be considered as a supplement to long-term culture media. Lastly, the study suggests that a co-culture of PSC-derived scHSCs and scLSECs improves the development of cell-specific phenotypes and functions, casting a positive outlook on the prospect of PSC-derived multicellular disease models.

## Data Availability

The original contributions presented in the study are included in the article/Supplementary Material, further inquiries can be directed to the corresponding author.

## Supplementary material

Supplementary Materials Table 3.

Supplementary Figures S1-S8 Data Sheet 1.

Supplementary Methods S1-S3 Data Sheet 2.
