# Supplementary material for: Characterization of human stem cell-derived hepatic stellate cells and liver sinusoidal endothelial cells during extended in vitro culture
Source: Front Bioeng Biotechnol. 2023 Jul 25;11:1223737. doi: 10.3389/fbioe.2023.1223737 (PMC10408301; doi:10.3389/fbioe.2023.1223737)
Supplement: Supplementary file 1 [file DataSheet2.pdf]

Supplementary Information

Document S2: Methods S1-S3

Characterization of Human Stem Cell-Derived Hepatic Stellate Cells and Liver Sinusoidal Endothelial Cells During Extended *in vitro* Culture

Ingrid Wilhelmsen[1,2*], *Mikel Amirola Martinez*[2*], *Justyna Stokowiec*[2], *Chencheng Wang*[2], *Aleksandra Aizenshtadt*[2**], *Stefan Krauss*[1,2**]

[1] Department of Immunology and Transfusion Medicine, Oslo University Hospital, Oslo, Norway.
[2] Hybrid Technology Hub - Centre of Excellence, Institute of Basic Medical Sciences, University of Oslo, Oslo, Norway.
*shared first authorship, **shared senior authorship

Methods S1. Quantification of α-SMA intensity in scHSCs and pHSCs.

Schematic flow chart of the workflow of the α-SMA intensity analysis. The script to the analysis is provided on GitHub: https://github.com/ingridwilhelmsen/a-SMA_analysis

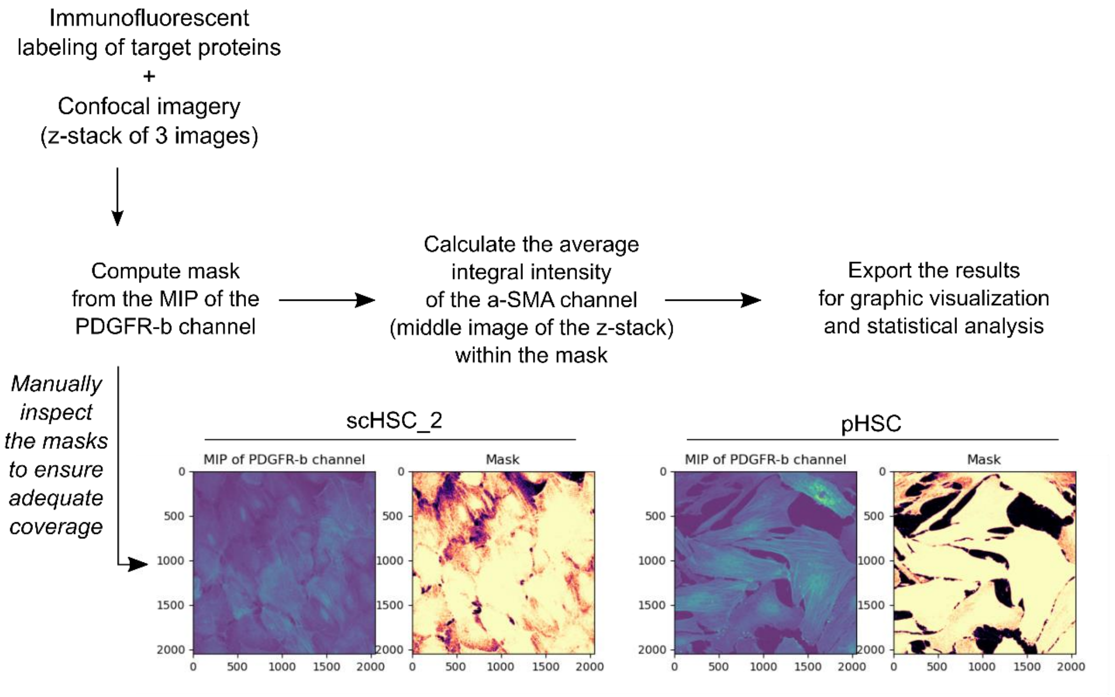

Methods S2. Quantification of UV signal in scHSCs and pHSCs.

Schematic flow chart of the workflow of the UV signal analysis and the automated macros used for analysis in ImageJ (Java 8 32-bit).

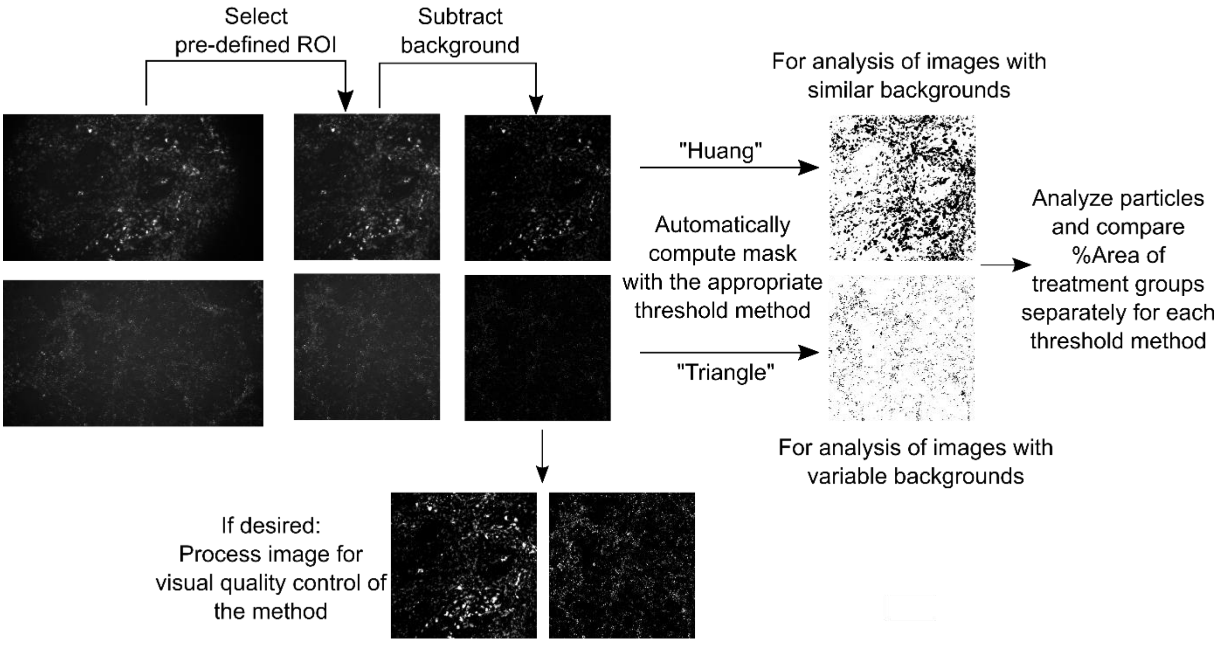

UV signal: “Huang” threshold macro

```
roiManager("Select", 0);
run("Duplicate...", " ");

setOption("ScaleConversions", true);
run("8-bit");
run("Subtract Background...", "rolling=25");
setAutoThreshold("Huang dark no-reset");
run("Analyze Particles...", "size=10-Infinity show=Masks display
clear summarize overlay");
close();
close();
```

UV signal: “Triangle” threshold macro

```
roiManager("Select", 0);
run("Duplicate...", " ");

setOption("ScaleConversions", true);
run("8-bit");
run("Subtract Background...", "rolling=25");
setAutoThreshold("Triangle dark no-reset");
run("Analyze Particles...", "size=0-Infinity show=Masks display
clear summarize overlay");
close();
close();
close();
```

Methods S3. Quantification of senescence signal in scHSCs and pHSCs.

The automated macro used for analysis of senescence signal.

Senescence signal: Macro

```
run("Split Channels");

close();
close();

roiManager("Select", 0);
run("Duplicate...", " ");

setAutoThreshold("Yen no-reset");
run("Analyze Particles...", "  show=Masks display clear summarize
overlay");

close();
close();
close();
```
