# Supplementary material for: Characterization of human stem cell-derived hepatic stellate cells and liver sinusoidal endothelial cells during extended in vitro culture
Source: Front Bioeng Biotechnol. 2023 Jul 25;11:1223737. doi: 10.3389/fbioe.2023.1223737 (PMC10408301; doi:10.3389/fbioe.2023.1223737)
Supplement: Supplementary file 4 [file DataSheet1.pdf]

Supplementary Information

Document S1: Figures S1-S8

Characterization of Human Stem Cell-Derived Hepatic Stellate Cells and Liver Sinusoidal Endothelial Cells During Extended *in vitro* Culture

Ingrid Wilhelmsen[1,2], Mikel Amirola Martinez[2*], Justyna Stokowiec[2], Chencheng Wang[2], Aleksandra Aizenshtadt[2**], Stefan Krauss[1,2**]*

[1] Department of Immunology and Transfusion Medicine, Oslo University Hospital, Oslo, Norway.

[2] Hybrid Technology Hub - Centre of Excellence, Institute of Basic Medical Sciences, University of Oslo, Oslo, Norway.

*shared first authorship, **shared senior authorship

Supplementary figure legends

Figure S1. (Related to Figure 1). Characterization of scHSCs.

(A) Representative bright-field images of scHSCs. Scale bars: 100 µm.
(B) Representative immunofluorescence confocal images of PDGFR-β, Vimentin, and NCAM1 in scHSCs. Scale bars: 40 µm.
(C) Representative graphs of flow cytometry analysis of surface marker PDGFR-β in scHSCs. AB: Antibody. N = 2 technical replicates.
(D) Representative immunofluorescence confocal images of PDGFR-β, α-SMA, and COL1α1 in scHSCs either treated or not treated with TGF-β. Scale bars: 40 µm.
(E) Representative bright-field and UV images of scHSCs either treated or not treated with TGF-β. Scale bars: 100 µm.
(F) Vitamin A storage of scHSCs indirectly measured by quantifying UV positivity, comparing cells either treated or not treated with TGF-β. N = 3 technical replicates.

Figure S2. (Related to Figures 2, 4, and 5). Characterization of scLSECs and scLSEC flow cytometry data.

(A) Representative bright-field images of cells differentiating from PSCs to scLSEC and pLSECs. Dark round circumferences in the "Expansion Passage 1" images correspond to residual Dyna beads used for cell-sorting of CD36+ cells. The length of the scale bars is specified in the individual images.
(B) Representative graphs of flow cytometry analysis of LYVE1 and *E.coli* bioparticles as well as VE-cadherin and AcLDL in scLSECs. Green: HUVEC controls, red: scLSEC on day 0 (the start of the long-term culture), blue: scLSECs on day 14 of the long-term culture, yellow: scLSECs treated with DAPT on day 14 of the long-term culture. N = 3 technical replicates.

Figure S3. (Related to Figure 3). Gene expression of scHSCs and pHSCs during long-term culture.

(A) Gene expression analysis of HSC markers (*ALCAM*, *DESMIN*, *LRAT*, *NCAM1*, *PDGFR-β*), fibrosis markers (*ACTA2*, *COL1α1*, *LOXL2*, *SMAD7*, *TIMP1*), and zonation markers (zone 1: *IGFBP3*, *NGFR*, *RGS4*, *TAGLN*; zone 2: *ADAMTSL2*, *PODN*, *RSPO3*, SPON2). Expression values were normalized to day 0 of the long-term culture. N = 3 technical replicates.

Figure S4. (Related to Figure 3). Characterization of scHSCs and pHSCs during long-term culture.

(A) Representative bright-field images of scHSCs and pHSCs during the long-term culture either treated or not treated with TGF-β. Scale bars: 100 µm.
(B) Representative bright-field images after histochemical senescence staining of scHSCs on day 14 (stains β-galactosidase blue, which is detectable in senescent cells). Scale bars: 100 µm.

(C) Representative bright-field and UV images of scHSCs and pHSC on day 5 either treated or not treated with TGF-β. Scale bars: 100 µm.
(D) Representative immunofluorescence confocal images of PDGFR-β, α-SMA, and COL1α1 in scHSCs on days 5 and 14. scHSCs were either treated or not treated with TGF-β. Scale bars: 40 µm.

Figure S5. (Related to Figure 4). Characterization of scLSECs during long-term culture.

(A) Relative gene expression levels of LSEC markers (*LYVE1*, *ICAM1*, *F8*, *PECAM1*, *PLVAP*, *CD36*), fibrosis markers (*CD34*, *COL5α2,* and *FBN1*), zone 1 (*Dll4*, *EphB2*, *NTN4*, *LTBP4,* and *MSR1*) and zone 3 (*KIT*, *CDH13,* and *THBD*) in scLSEC_1 and scLSEC_2. Expression levels were measured on days 0, 5, and 14. Values of expression were normalized to day 0 values. N = 3 technical replicates.
(B) Representative bright-field images of scLSECs at the start (day 0) and the end (day 14) of the long-term culture. Scale bars: 100 µm.
(C) Representative immunofluorescence confocal images of LYVE1 and VE-cadherin in scLSEC_1 on days 5 and 14. Scale bars: 20 µm.

Figure S6. (Related to Figure 5). Characterization of the effect of Notch inhibition on scHSCs and scLSECs.

(A) Gene expression of Notch signaling pathway-associated genes *JAG1*, *HEY1*, and *HES1* in scHSCs and scLSECs grown in untreated medium and medium supplemented with DAPT. The samples were harvested on day 14 and expression values were normalized to the untreated samples. For scHSCs: n = 4 independent differentiations of four different PSC lines with 3 technical replicates per differentiation, for scLSECs: n = 2 independent differentiations of two different PSC lines with N = 3 technical replicates per differentiation. All technical replicates are shown.
(B) Gene expression of fibrosis-associated markers after treatment with TGF-β in pHSCs. Expression values were normalized to the control samples. N = 3 technical replicates.
(C) Vitamin A storage of scHSCs indirectly measured by quantifying UV positivity, comparing cells grown in untreated medium and medium supplemented with DAPT. N = 3 technical replicates.
(D) Representative senescence-stained images of scHSCs and quantification of the senescence signal on day 14 (stains β-galactosidase blue, which is detectable in senescent cells). Scale bars: 100 µm. n = 3 independent differentiations, N = 3 technical replicates per differentiation. All data points are shown
(E) Representative bright-field images of scLSECs grown in untreated medium and medium supplemented with DAPT on day 14 of the culture, the latter group showing branch-like condensations. Scale bars: 100 µm.

Data from scHSC_1 and scLSEC_1 (hESC-derived) are colored purple or shown as purple dots throughout the figure.

Figure S7. (Related to Figure 5). Immunofluorescence images of scHSCs and pHSCs under Notch Inhibition.

(A) Representative immunofluorescence confocal images of PDGFR-β, α-SMA, and COL1α1 in scHSCs and pHSCs. The cells were grown in a medium supplemented with DAPT and a pro-fibrotic stimulus was induced by TGF-β. Scale bars: 40 µm.

(B) Representative immunofluorescence confocal images of LYVE1 and VE-cadherin in scLSEC_2 grown in untreated medium and medium supplemented with DAPT on day 14 of the culture. Scale bars: 20 µm.

Figure S8. (Related to Figure 6). Co-culture of scHSCs and scLSECs.

(A) Gene expression profile of scHSCs and scLSECs grown in co-culture for 14 days, testing HSC markers (*ALCAM*, *DESMIN*, *LRAT*, *NCAM1*, *PDGFR-β*), HSC fibrosis markers (*ACTA2*, *COL1α1*, *LOXL2*, *SMAD7*, *TIMP1*) and HSC zonation markers (zone 1: *IGFBP3*, *NGFR*, *RGS4*, *TAGLN*; zone 2: *ADAMTSL2*, *PODN*, *RSPO3*, SPON2), LSEC markers (*CD36*, *F8*, *LYVE1*, *PLVAP*), LSEC fibrosis markers (*COL5α2*, *FBN1*, *CD34*), and LSEC zonation markers (zone 1: *DLL4*; zone 3: *CDH13*). N = 3 technical replicates.

(B) Comparing vitamin A storage measured by UV signal in scHSCs grown in monoculture either with HSC expansion medium or HSC-LSEC co-culture medium for 14 days (see table 2 for medium compositions). N = 3 technical replicates, each replicate is the average of 5 independent fields.

S1

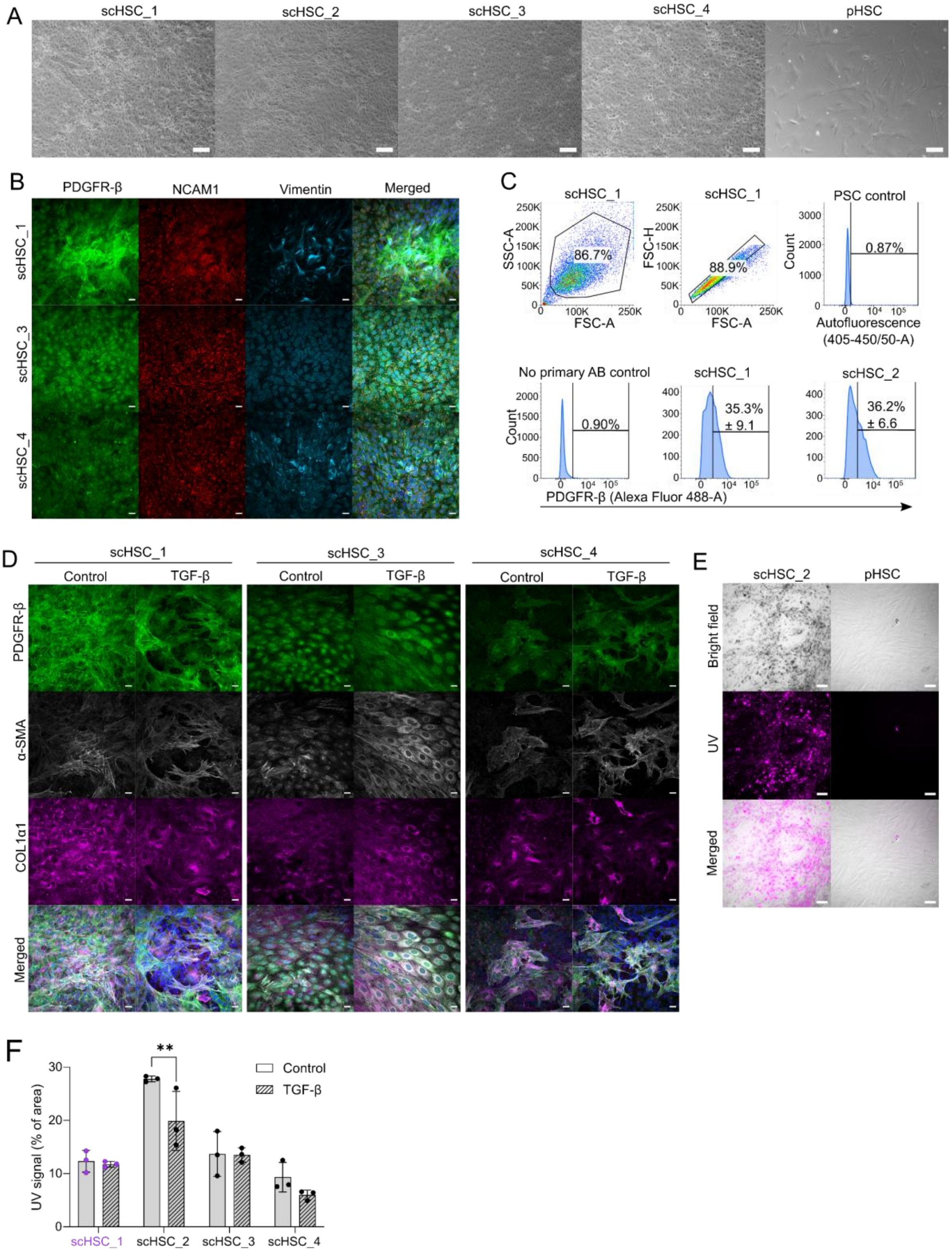
A
scHSC_1
scHSC_2
scHSC_3
scHSC_4
pHSC
B
PDGFR-β
NCAM1
Vimentin
Merged
scHSC_1
scHSC_3
scHSC_4
C
scHSC_1
SSC-A
FSC-A
86.7%
scHSC_1
FSC-H
FSC-A
88.9%
PSC control
Count
0.87%
Autofluorescence (405-450/50-A)
No primary AB control
Count
0.90%
scHSC_1
35.3% ± 9.1
scHSC_2
36.2% ± 6.6
PDGFR-β (Alexa Fluor 488-A)
D
scHSC_1
scHSC_3
scHSC_4
Control
TGF-β
PDGFR-β
α-SMA
COL1α1
Merged
E
scHSC_2
pHSC
Bright field
UV
Merged
F
UV signal (% of area)
**
Control
TGF-β
scHSC_1
scHSC_2
scHSC_3
scHSC_4

S2

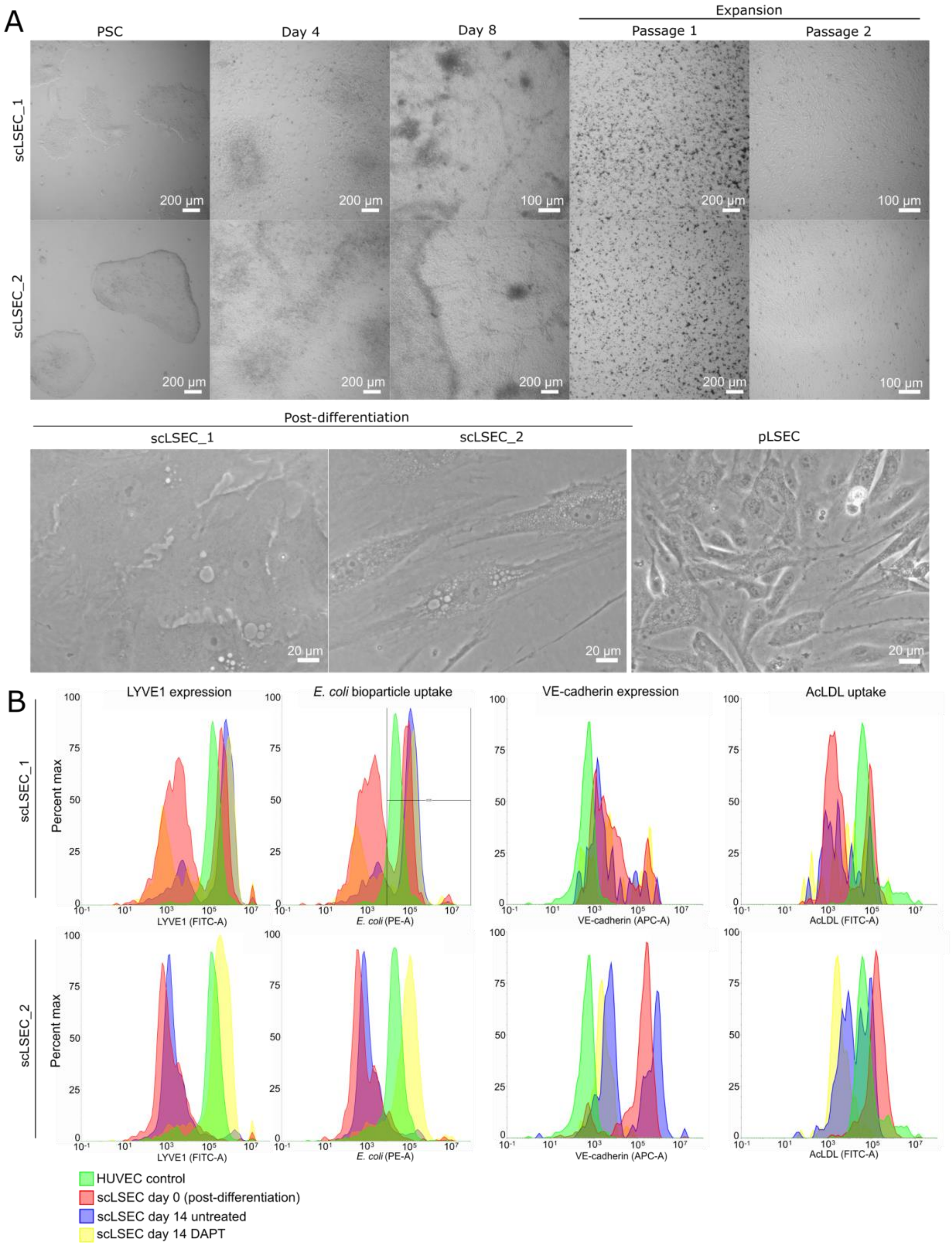
A
PSC
Day 4
Day 8
Expansion
Passage 1
Passage 2
scLSEC_1
scLSEC_2
200 µm
100 µm
Post-differentiation
scLSEC_1
scLSEC_2
pLSEC
20 µm
B
LYVE1 expression
E. coli bioparticle uptake
VE-cadherin expression
AcLDL uptake
Percent max
LYVE1 (FITC-A)
E. coli (PE-A)
VE-cadherin (APC-A)
AcLDL (FITC-A)
HUVEC control
scLSEC day 0 (post-differentiation)
scLSEC day 14 untreated
scLSEC day 14 DAPT

S3

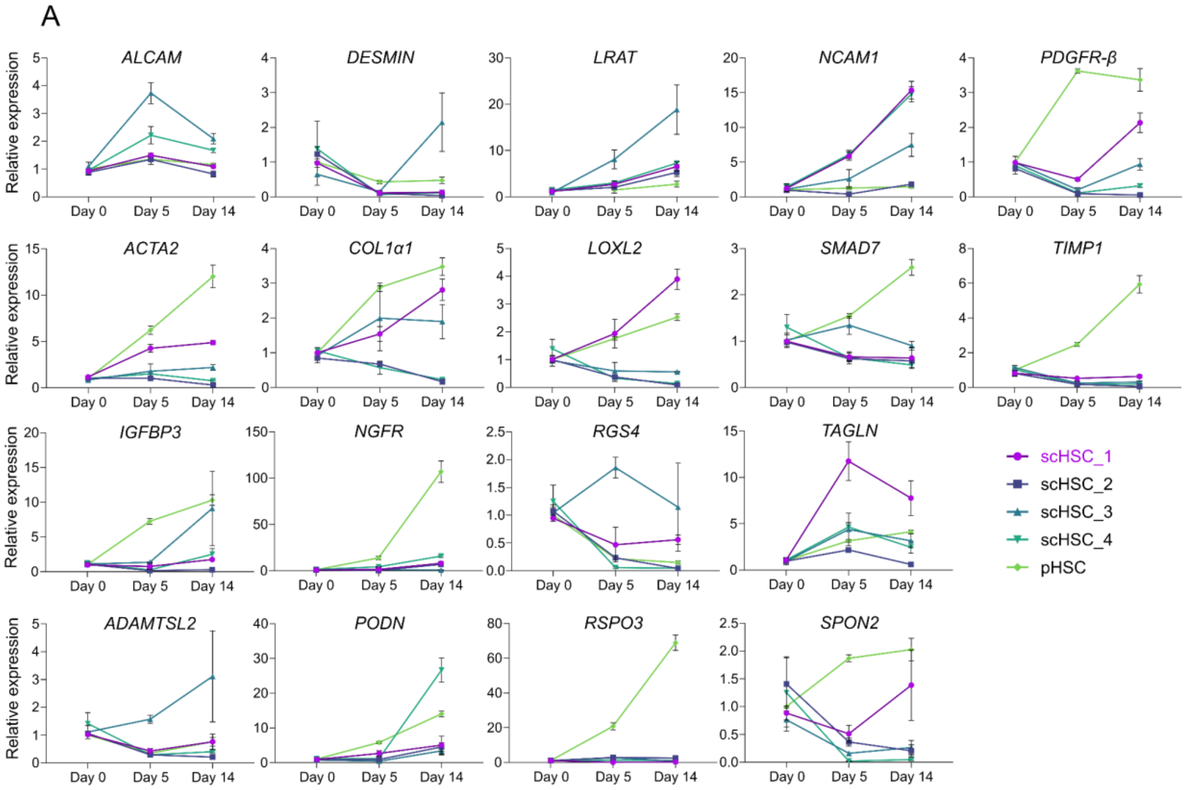

A
Relative expression
ALCAM
DESMIN
LRAT
NCAM1
PDGFR-β
ACTA2
COL1α1
LOXL2
SMAD7
TIMP1
IGFBP3
NGFR
RGS4
TAGLN
ADAMTSL2
PODN
RSPO3
SPON2
Day 0
Day 5
Day 14
scHSC_1
scHSC_2
scHSC_3
scHSC_4
pHSC

S4

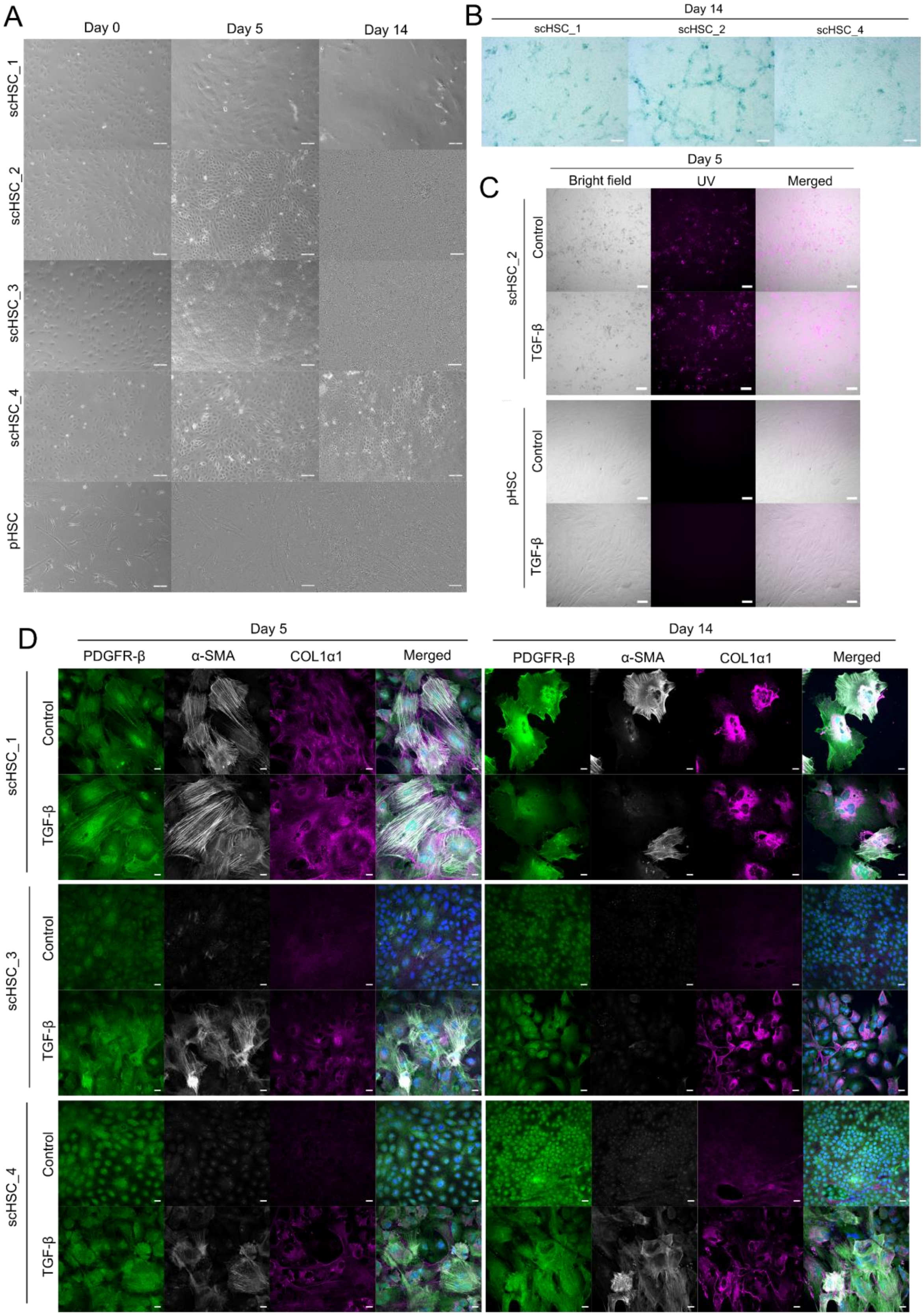

A
Day 0
Day 5
Day 14
scHSC_1
scHSC_2
scHSC_3
scHSC_4
pHSC
B
Day 14
scHSC_1
scHSC_2
scHSC_4
C
Day 5
Bright field
UV
Merged
scHSC_2
Control
TGF-β
pHSC
Control
TGF-β
D
Day 5
Day 14
PDGFR-β
α-SMA
COL1α1
Merged
PDGFR-β
α-SMA
COL1α1
Merged
scHSC_1
Control
TGF-β
scHSC_3
Control
TGF-β
scHSC_4
Control
TGF-β

S5

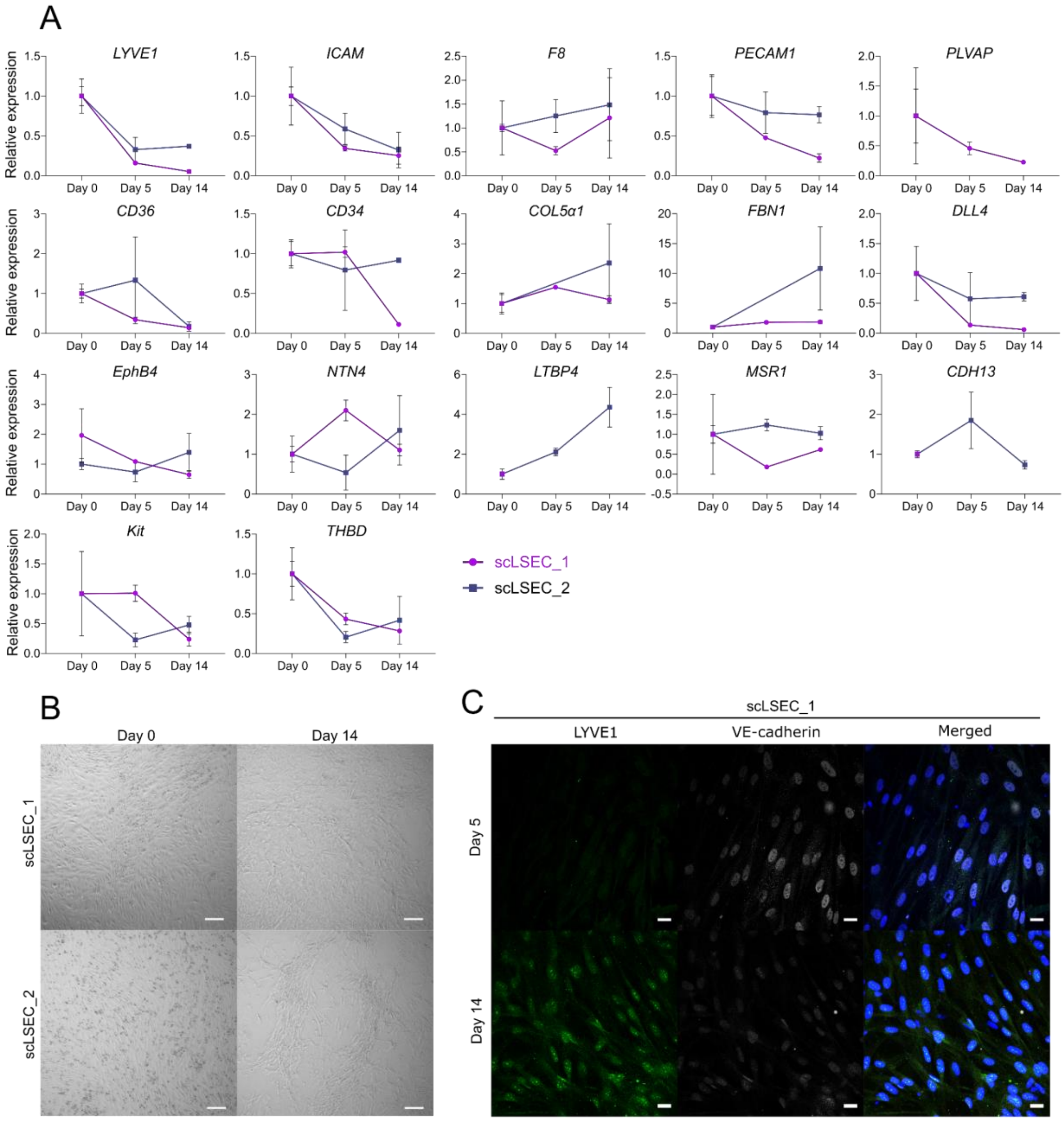

A
LYVE1
ICAM
F8
PECAM1
PLVAP
CD36
CD34
COL5α1
FBN1
DLL4
EphB4
NTN4
LTBP4
MSR1
CDH13
Kit
THBD
Relative expression
Day 0
Day 5
Day 14
scLSEC_1
scLSEC_2
B
Day 0
Day 14
scLSEC_1
scLSEC_2
C
scLSEC_1
LYVE1
VE-cadherin
Merged
Day 5
Day 14

S6

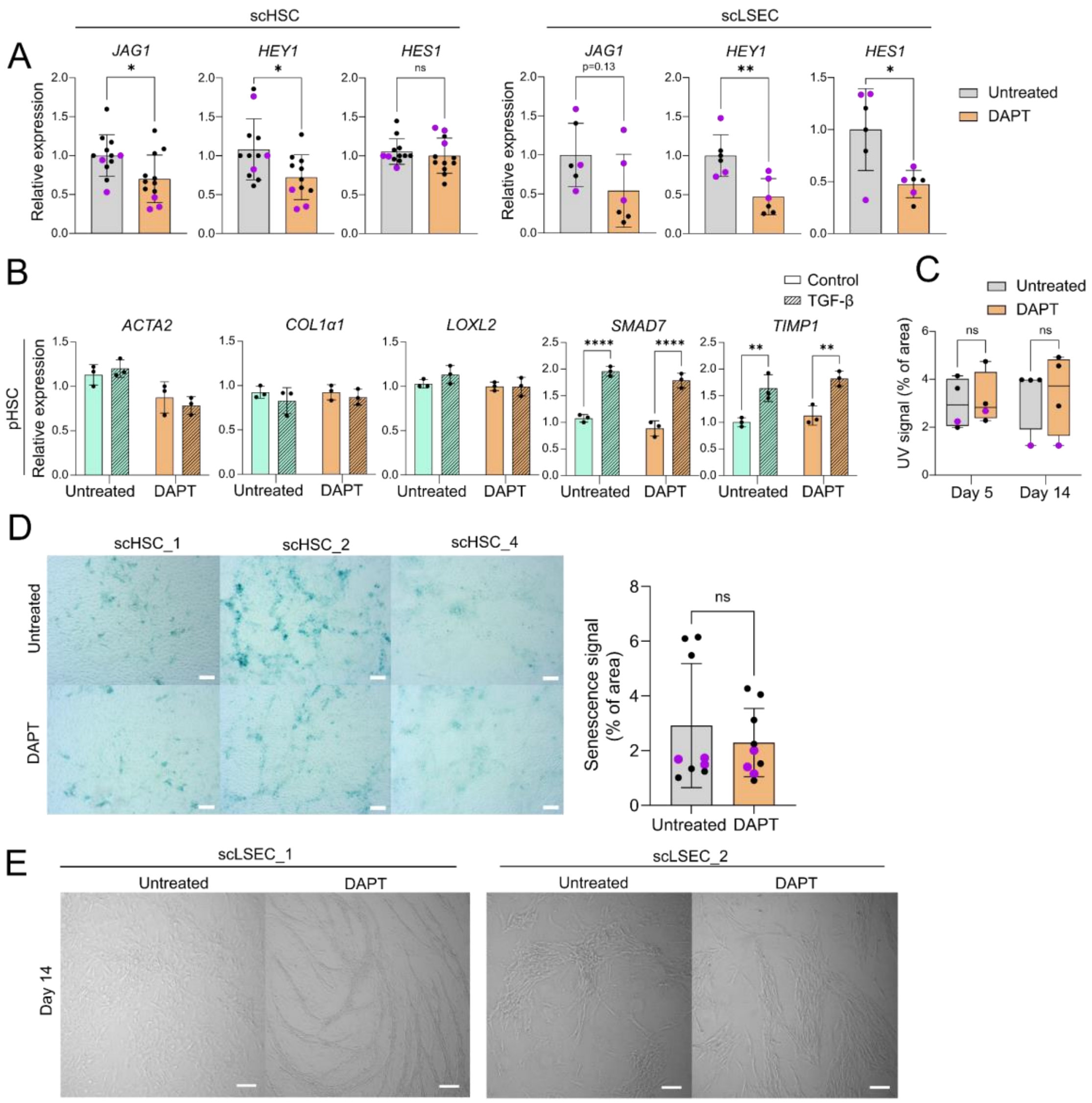

scHSC
scLSEC
A
JAG1
HEY1
HES1
JAG1
p=0.13
HEY1
HES1
Relative expression
Untreated
DAPT
B
Control
TGF-β
ACTA2
COL1α1
LOXL2
SMAD7
TIMP1
pHSC
Relative expression
Untreated
DAPT
C
UV signal (% of area)
Day 5
Day 14
D
scHSC_1
scHSC_2
scHSC_4
Untreated
DAPT
Senescence signal (% of area)
E
scLSEC_1
scLSEC_2
Day 14

S7

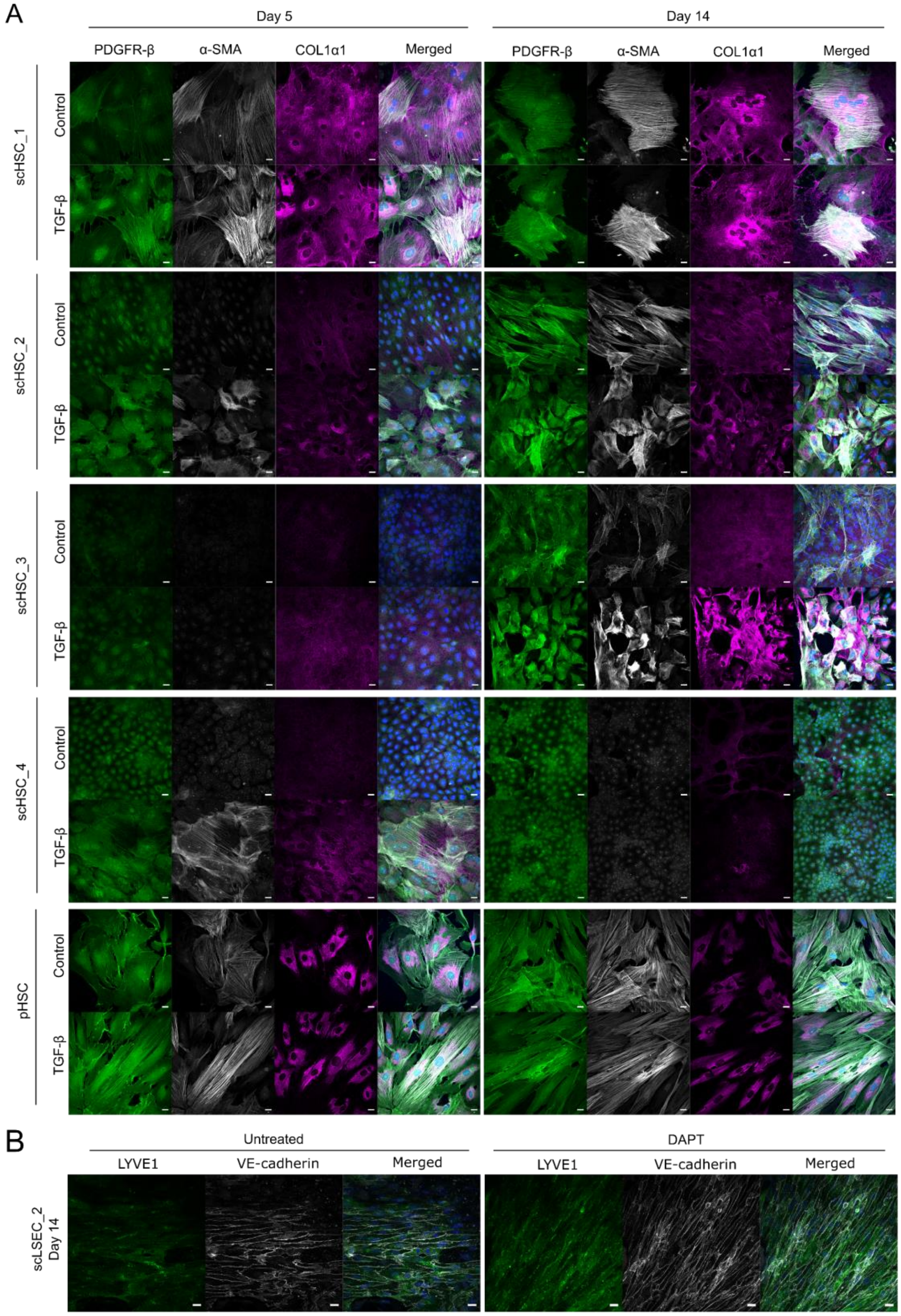
A
Day 5
Day 14
PDGFR-β
α-SMA
COL1α1
Merged
PDGFR-β
α-SMA
COL1α1
Merged
scHSC_1
Control
TGF-β
scHSC_2
Control
TGF-β
scHSC_3
Control
TGF-β
scHSC_4
Control
TGF-β
pHSC
Control
TGF-β
B
Untreated
DAPT
LYVE1
VE-cadherin
Merged
LYVE1
VE-cadherin
Merged
scLSEC_2
Day 14

S8

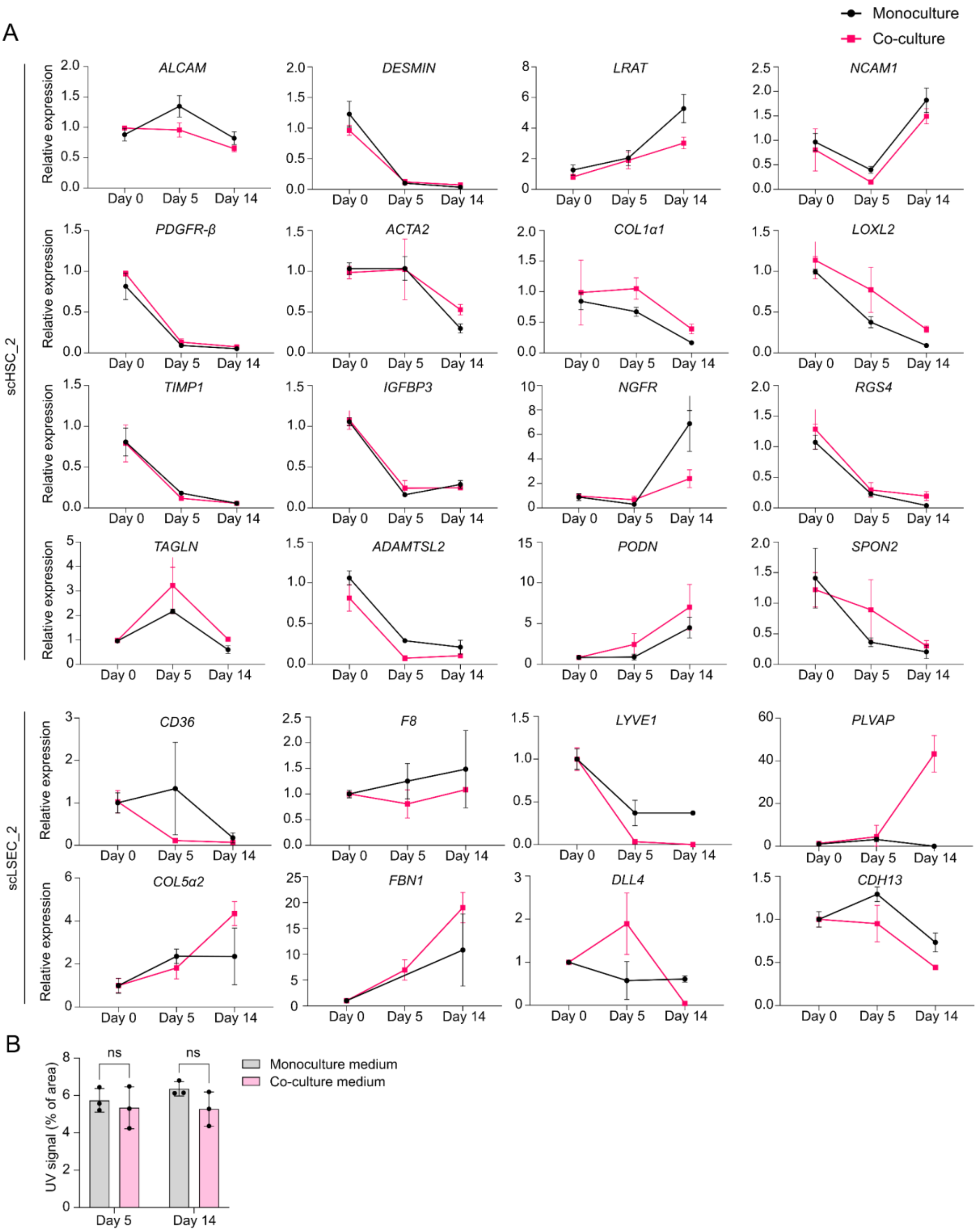
A
Monoculture
Co-culture
scHSC_2
scLSEC_2
Relative expression
ALCAM
DESMIN
LRAT
NCAM1
PDGFR-β
ACTA2
COL1α1
LOXL2
TIMP1
IGFBP3
NGFR
RGS4
TAGLN
ADAMTSL2
PODN
SPON2
CD36
F8
LYVE1
PLVAP
COL5α2
FBN1
DLL4
CDH13
Day 0
Day 5
Day 14
B
UV signal (% of area)
ns
Monoculture medium
Co-culture medium
